# APC/C^Cdh1^ Enables Removal of Shugoshin-2 from the Arms of Bivalent Chromosomes by Moderating Cyclin-Dependent Kinase Activity

**DOI:** 10.1016/j.cub.2017.04.023

**Published:** 2017-05-22

**Authors:** Ahmed Rattani, Randy Ballesteros Mejia, Katherine Roberts, Maurici B. Roig, Jonathan Godwin, Michael Hopkins, Manuel Eguren, Luis Sanchez-Pulido, Elwy Okaz, Sugako Ogushi, Magda Wolna, Jean Metson, Alberto M. Pendás, Marcos Malumbres, Béla Novák, Mary Herbert, Kim Nasmyth

**Affiliations:** 1 Department of Biochemistry, University of Oxford, South Parks Road, Oxford OX1 3QU, UK; 2 Newcastle Fertility Centre, Centre for Life, Times Square, Newcastle upon Tyne NE1 4EP, UK; 3 Wellcome Trust Centre for Mitochondrial Research, Institute for Genetic Medicine, Newcastle University, Newcastle upon Tyne NE4 5PL, UK; 4 Cell Division and Cancer Group, Spanish National Cancer Research Center (CNIO), 28029 Madrid, Spain; 5 MRC Human Genetics Unit, Institute of Genetics and Molecular Medicine, University of Edinburgh, Edinburgh EH4 2XU, UK; 6 Instituto de Biología Molecular y Celular del Cáncer de Salamanca, CSIC-Universidad de Salamanca, 37007 Salamanca, Spain

**Keywords:** APC/C^Cdh1^, shugoshin-2, aurora kinase, cohesin, meiosis, aneuploidy

## Abstract

In mammalian females, germ cells remain arrested as primordial follicles. Resumption of meiosis is heralded by germinal vesicle breakdown, condensation of chromosomes, and their eventual alignment on metaphase plates. At the first meiotic division, anaphase-promoting complex/cyclosome associated with Cdc20 (APC/C^Cdc20^) activates separase and thereby destroys cohesion along chromosome arms. Because cohesion around centromeres is protected by shugoshin-2, sister chromatids remain attached through centromeric/pericentromeric cohesin. We show here that, by promoting proteolysis of cyclins and Cdc25B at the germinal vesicle (GV) stage, APC/C associated with the Cdh1 protein (APC/C^Cdh1^) delays the increase in Cdk1 activity, leading to germinal vesicle breakdown (GVBD). More surprisingly, by moderating the rate at which Cdk1 is activated following GVBD, APC/C^Cdh1^ creates conditions necessary for the removal of shugoshin-2 from chromosome arms by the Aurora B/C kinase, an event crucial for the efficient resolution of chiasmata.

## Highlights

• Sgol2 phosphorylation by Aurora B/C kinase dissociates it from chromosome arms
• APC/C^Cdh1^ ensures a gradual increase in Cdk1 activity at the resumption of meiosis
• APC/C^Cdh1^ activity enables Aurora B/C kinase-dependent Sgol2 phosphorylation
• By promoting Sgol2 removal from chromosome arms, APC/C^Cdh1^ prevents non-disjunction

## Graphical Abstract

**Figure:**
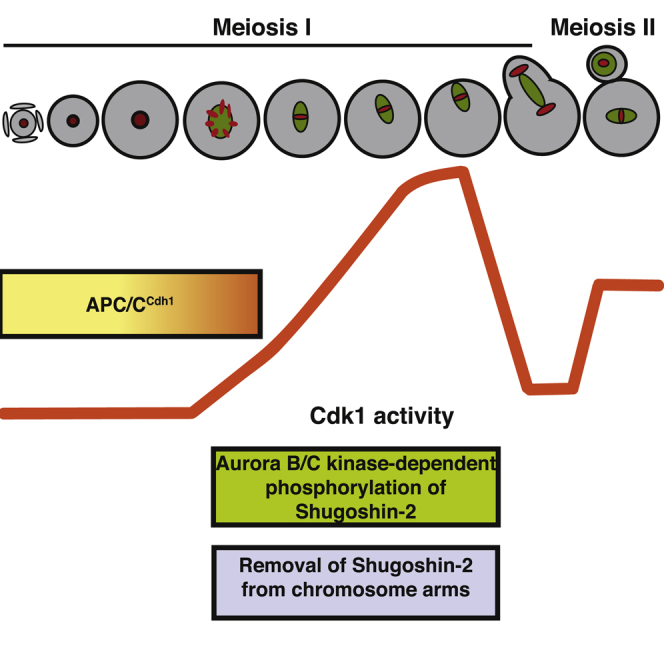

## Teaser

Rattani et al. report that the APC/C^Cdh1^ moderates the rise in Cdk1 activity as mouse oocytes resume meiosis. This creates a window of low Cdk1 activity shortly after GVBD, which promotes Aurora B/C kinase-dependent removal of shugoshin-2 from chromosome arms, failure of which could result in aneuploidy at the first meiotic division.

## Introduction

In mitotic cells, each round of chromosome segregation is preceded by DNA replication, and as a result, chromosome numbers remain constant during cell proliferation. Meiosis, in contrast, involves two rounds of chromosome segregation (meiosis I and II) following only a single round of DNA replication and therefore produces haploid gametes with only a single set of chromosomes from diploid germ cells containing complete sets of both paternal and maternal chromosomes [1].

Another major difference occurs specifically in female germ cells in vertebrates, including all mammals. In mitotic cells, there is usually only a short gap (known as G2) between the completion of DNA replication and the onset of chromosome segregation, which is initiated by a rapid rise in Cdk1 kinase activity. In female mammals, DNA replication and the recombination between non-sister homologous chromatids that creates bivalent chromosomes is completed by the time of birth, but chromosome segregation only takes place periodically upon sexual maturation, which may be many years later [1].

Thus, following recombination, primordial germ cells surrounded by a single layer of supporting follicular cells arrest for extended periods of time in prophase of meiosis I with low levels of Cdk1 activity associated with mitotic cyclins. These primordial follicles enter a period of growth to become prophase arrested (the germinal vesicle [GV] stage) oocytes but can only resume meiosis and enter prometaphase I when activated by luteinizing hormone [2] or when released from follicular cells in vitro [3]. As occurs during mitosis, a rise in Cdk1 activity accompanies entry into prometaphase I [4], but curiously this rise is not as abrupt as in mitotic cells [5, 6, 7].

Two processes regulate Cdk1 activity during the prophase I arrest of oocytes: association with cyclins and phosphorylation by the Wee1 protein kinase [8, 9]. The latter inhibits Cdk1 activity, even during their growth phase [8]. Its eventual reversal by the phosphatase Cdc25B accompanies and is required for germinal vesicle breakdown (GVBD) and the onset of chromosome condensation [10]. Cdk1 is, however, only active when bound to cyclins, whose abundance is therefore also a crucial factor [11]. A key determinant of their levels is the rate of degradation, a process controlled through ubiquitinylation by the anaphase-promoting complex or cyclosome (APC/C) [12, 13, 14]. A major increase in the rate of degradation mediated by APC/C associated with its activator Cdc20 (APC/C^Cdc20^) takes place when all bivalents co-orient on the meiotic spindle, which turns off production of the inhibitory mitotic checkpoint complex (MCC) [15]. This leads to a drop in Cdk1 activity and in the levels of securin, events that lead to activation of separase and thereby cleavage of cohesin holding bivalent chromosomes together [16, 17, 18].

Less well understood is the role of other Cdc20-like APC/C activator proteins. Best characterized is the role of *S. cerevisiae*’s Ama1 [19]. This meiosis-specific WD40 protein generates a form of the APC/C (APC/C^Ama1^) that helps maintain a prophase-like state by preventing premature accumulation of B-type cyclins and a transcription factor that promotes expression of mitotic cyclins [20]. An analogous form of regulation exists in mammals, where APC/C^Cdh1^ degrades cyclin B during prophase I arrest, blocking entry into metaphase I [21, 22]. Recent work has shown that APC/C^Cdh1^ also has a key role in maintaining the prolonged prophase arrest of primordial follicles [23], though how it does so remains unclear, as is the interplay between cyclin degradation and Wee1-mediated Cdk1 phosphorylation.

What is clear is that Cdk1 activation is essential for GVBD, chromosome condensation, and the alignment of bivalents on meiotic spindles [4, 6, 24]. During this process, the Aurora B kinase eliminates kinetochore-microtubule attachments that fail to give rise to tension, whereas the spindle assembly checkpoint (SAC) prevents premature activation of APC/C^Cdc20^ [25, 26, 27]. Only when all bivalents have co-oriented, with maternal and paternal kinetochores pulled in opposite directions, is production of the MCC switched off [28]. This induces destruction of B-type cyclins and securin by APC/C^Cdc20^ and thereby activation of separase, which triggers the resolution of chiasmata by cleaving cohesin’s Rec8 subunit along chromosome arms [17, 29, 30]. Crucially, cohesin in the vicinity of centromeres is protected from separase during meiosis I by protein phosphatase 2A (PP2A) bound to the shugoshin-like protein 2 (Sgol2) [31]. By holding chromatids together after meiosis I, centromeric cohesion ensures that dyads and not individual chromatids are generated at meiosis I, which makes possible the bi-orientation of sister kinetochores during meiosis II [32]. Subsequent destruction of centromeric cohesion when separase is re-activated upon fertilization triggers the eventual disjunction of individual chromatids and the creation of haploid gametes.

Using female-germ-cell-specific *Cdh1* [33], *Apc2* [34], and *Cdc20* [35] knockouts, we show here that APC/C^Cdh1^ ensures that meiotic resumption is triggered by and/or accompanied by only a modest increase in Cdk1 activity. This creates a window following GVBD during which the Aurora B/C kinase can promote removal of Sgol2 from chromosome arms and its accumulation at centromeres. By jeopardizing the resolution of chiasmata, a lack of APC/C^Cdh1^ greatly increases non-disjunction at the first meiotic division. Our finding challenges the preconception that a step change in Cdk1 activity is sufficient to orchestrate chromosome segregation when cells enter M phase [36] but rather reveals that a gradual increase in Cdk1 activity ensures balanced segregation of chromosomes at the first meiotic division.

## Results

### By Destroying Cyclins and Cdc25B, APC/C^Cdh1^ Orchestrates a Gradual Increase in Cdk1 Activity after GVBD

Cdh1 knockdown at the GV stage increases non-disjunction of chromosomes at the first meiotic division [37]. However, at the metaphase to anaphase transition, APC/C conjugates with Cdc20 (APC/C^Cdc20^) to activate separase and bring about segregation of chromosomes [29]. Therefore, to investigate the role of APC/C^Cdh1^ at the first meiotic division, we deleted *Apc2* and *Cdh1* specifically from germ cells by crossing *Apc2*^*f/f*^ and *Cdh1*^*f/f*^ females with transgenic males expressing Cre recombinase under either growth-differentiation-factor-9-promoter-mediated promoter (*Gdf9-iCre*), expressed in all oocytes from day 3 after birth [38], or zona pellucida 3 promoter (*Zp3-Cre*), expressed only after oocytes enter the growth phase. As in the case of *Apc2*^*f/*f^ *Zp3Cre* and *Cdc20*^*f/f*^ *Zp3Cre* [26, 29], *Apc2*^*f/f*^ *Gdf9-iCre* females were completely infertile. In contrast, *Cdh1*^*f/f*^ *Gdf9-iCre* females produced a litter at 6–12 weeks, albeit one with fewer pups than littermate controls. Despite this early fertility, all *Cdh1*^*f/f*^ *Gdf9-iCre* females were infertile by early adulthood (i.e., after 12 or 13 weeks) and had atrophied ovaries. Crucially, primordial follicles were rapidly depleted (unpublished data).

**Figure 1:**
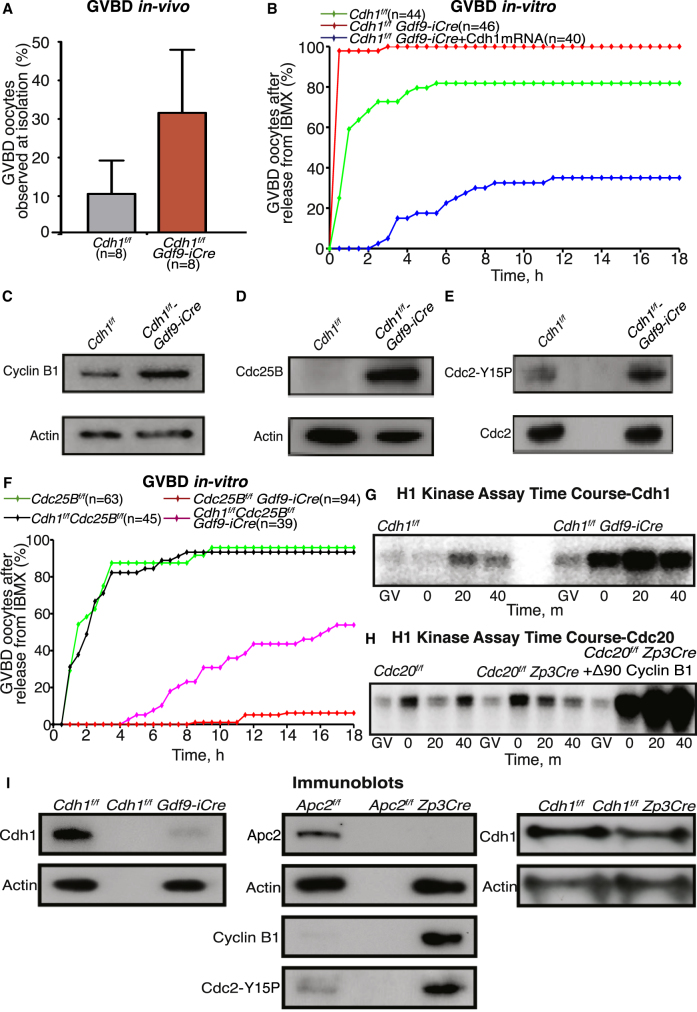
By Maintaining Low Levels of Cdc25B and Cyclin B1, the APC/C^Cdh1^ Maintains GV Arrest, Prevents Premature Entry into Meiosis, and Ensures a Gradual Increase in Cdk1 Activity after GVBD (A) Oocytes harvested from *Cdh1*^*f/f*^ and *Cdh1*^*f/f*^*Gdf9-iCre* ovaries in the presence of IBMX that had already resumed meiosis upon isolation were quantified. GVBD rates for each of the indicated genotypes are plotted as a percentage of the total oocytes observed. Mean and SDs are displayed, and the number of females used is indicated (n). (B) GV-stage oocytes harvested in the presence of IBMX were released into the M16 medium. The kinetics of GVBD was captured using a time-lapsed confocal microscope. Microinjections were performed at GV stage in the presence of IBMX. The number of oocytes analyzed is indicated (n). (C and D) GV-stage oocytes harvested from ovaries isolated from 4-week-old females were immunoblotted for cyclin B1 (C) and Cdc25B (D). Actin was used as loading control. One hundred twenty GV-stage oocytes were pooled for each lane. (E) Fully grown GV-arrested oocytes were immunoblotted for phospho-Cdk1 (Tyr15) and Cdk1. For each lane, 120 GV-stage oocytes were pooled. (F) GV-stage oocytes harvested from the indicated genotypes were imaged for 18 hr. The kinetics of GVBD is displayed. The number of oocytes imaged is indicated (n). (G and H) Cdk1 kinase activity was estimated using in vitro phosphorylation of histone H1. Oocytes from *Cdh1*^*f/f*^ and *Cdh1*^*f/f*^*Gdf9-iCre* (G) and *Cdc20*^*f/f*^, *Cdc20*^*f/f*^*Zp3Cre*, and *Cdc20*^*f/f*^*Zp3Cre* microinjected with Δ90-cyclin B1 (H) ovaries at GV, GVBD, and 20 and 40 min post-GVBD were incubated with radiolabeled ATP and histone H1. Samples were resolved on SDS-PAGE gels, and incorporated radioactivity was imaged. (I) GV-stage oocytes from each of the indicated genotypes depicted were immunoblotted for Cdh1 or Apc2 to confirm depletion. Actin was used as a loading control. Extracts from *Apc2*^*f/f*^ and *Apc2*^*f/f*^*Zp3C*re were also immunoblotted for cyclin B1 and Cdc2-Y15P. For each lane, cell lysates from 120 GV-stage oocytes were loaded. Except for the Cdh1 Gdf9-iCre knockout and control ovaries, which were isolated at 4 weeks post-birth due to rapid depletion of the primordial follicles, ovaries from all other crosses were isolated at 6 weeks post-birth. For *Apc2* knockout results, see Figure S1.

Even though the conditional *Cdh1* knockout females quickly exhausted the primordial follicles, they still produced a few fully mature oocytes until about 7 weeks post-birth, though about one-third of those had undergone GVBD and resumed meiosis in vivo (Figure 1A). Even in the oocytes arrested at the GV stage by IBMX in vitro, onset of GVBD upon release was accelerated and more efficient when Cdh1 was depleted (Figure 1B). The fact that microinjection of Cdh1 mRNA into GV oocytes had the opposite effect implies that the APC/C^Cdh1^ is actively engaged in destroying substrates that promote GVBD in fully grown GV-arrested oocytes (Figure 1B).

Western blot analysis of cell lysates from the fully mature GV stage oocytes revealed that both cyclin B1 and Cdc25B were elevated in oocytes lacking Cdh1 (Figures 1C and 1D). These increases were accompanied by an increase in Cdk1-Y15P (Figure 1E), suggesting that many, but not all, excess cyclin B/Cdk1 complexes may be inhibited by Wee1-mediated Y15 phosphorylation. Furthermore, simultaneous depletion of Cdc25B in Cdh1 knockout ovaries greatly reduced and delayed GVBD in vitro (Figure 1F), suggesting that loss of Cdh1 accelerates GVBD by elevating Cdk1 activity.

To investigate the consequences of this enlarged pool of latent Cdk1, we measured its kinase activity in oocytes following GVBD in vitro, comparing those from *Cdh1*^*f/f*^ and *Cdh1*^*f/f*^ *Gdf9-iCre* females. This revealed that Cdh1 depletion increased by several-fold the rise in Cdk1 activity following GVBD. In control oocytes, only a modest increase in Cdk1 activity accompanies GVBD and levels continue to rise gradually (Figure 1G) [5, 6, 7]. In contrast, in Cdh1-depleted oocytes Cdk1 activity increases precipitously at GVBD and remains high (Figure 1G). Greater stability of cyclin B1 is presumably responsible for this phenomenon, as it can be reproduced merely by injecting oocytes lacking Cdc20 with mRNA encoding a non-degradable form of cyclin B1 (Δ90-cyclin B1; Figure 1H).

Studying the consequences of this major change in Cdk1 dynamics is greatly complicated by the depletion of primordial follicles in *Cdh1*^*f/f*^ *Gdf9-iCre* females, which therefore produce insufficient numbers of oocytes to study. To alleviate this problem, we used a different Cre-expressing transgene, namely *Zp3Cre*, which expresses Cre from the Zona pellucida 3 promoter only after commencement of the oocyte’s growing phase [38, 39]. We assumed that the depletion of primordial follicles observed in *Cdh1*^*f/f*^ *Gdf9-iCre* females is a developmental-stage-specific process and would not take place when Cdh1 is depleted solely during the growing phase. Unfortunately, oocytes from *Cdh1*^*f/f*^ *Zp3Cre* females still contain high levels of Cdh1 (Figure 1I), suggesting that its mRNA and/or protein is stable during the growing phase, even though this lasts over 2 weeks.

To circumvent our inability to deplete Cdh1 solely during the oocyte’s growing phase, we tested whether depletion of a core subunit of the APC/C, namely Apc2, would achieve the same goal. The gene products of the *Apc2* gene are more unstable than those of *Cdh1*, and western blotting revealed little or no Apc2 protein in GV-stage oocytes from *Apc2*^*f/f*^ *Zp3Cre* oocytes (Figure 1I). Indeed, it has already been established that these oocytes cannot destroy securin and cannot therefore undergo meiosis I [26]. Because APC/C^Cdc20^ has little or no role until the onset of cyclin B1 and securin proteolysis shortly before the first meiotic division and because Cdc20 and Cdh1 appear to be the sole accessory APC/C factors in mammals, Apc2 depletion should phenocopy that of Cdh1. Consistent with this notion, oocytes from *Apc2*^*f/f*^ *Zp3Cre* females contained high levels of cyclin B1 and Cdk1-Y15P at GV stage (Figure 1I). They also had a higher fraction of oocytes that had already undergone GVBD upon isolation (Figure S1A). Moreover, entry into meiosis was accelerated after release from IBMX arrest and oocytes resumed meiosis with persistently high Cdk1 activity (Figures S1B and S1C), comparable to that observed in oocytes from *Cdh1*^*f/f*^ *Gdf9-iCre* females. Because no such effect is observed in oocytes from *Cdc20*^*f/f*^ *Zp3Cre* females (Figure 1H), we assume that their high Cdk1 activity is due exclusively to loss of APC/C^Cdh1^ activity.

### Destruction of Cyclins by APC/C^Cdh1^ Facilitates Removal of Sgol2 from Chromosome Arms

**Figure 2:**
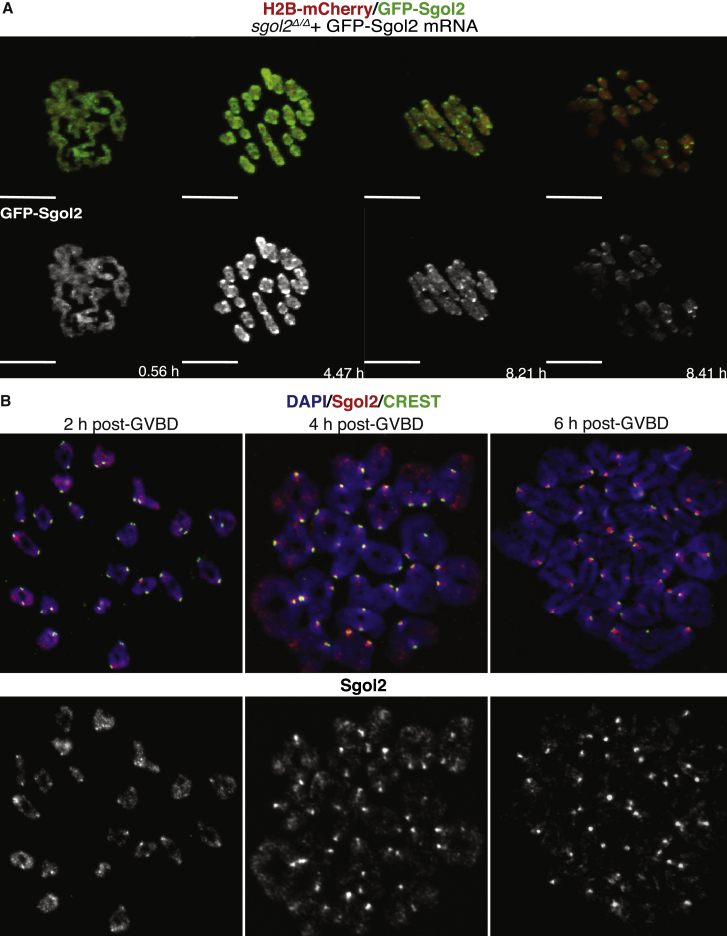
Following GVBD, Sgol2 Localizes on the Chromosome Arms and Kinetochores, and It Gradually Concentrates on Kinetochores during Late Metaphase (A) GV-stage oocytes harvested from *Sgol2*-deleted females were microinjected in M2 medium supplemented with IBMX with mRNA encoding GFP-Sgol2 and H2B-mCherry. After 1 hr of incubation, oocytes were released, and time-lapse confocal microscope images were captured for 12–14 hr following GVBD. Representative Z-projected images are displayed. (B) GV-stage oocytes harvested from wild-type control females were cultured for 2, 4, and 6 hr following GVBD. Chromosome spreads prepared at the indicated times following GVBD were stained for DNA (blue), Sgol2 (red), and CREST (green). See also Figure S2.

**Figure 3:**
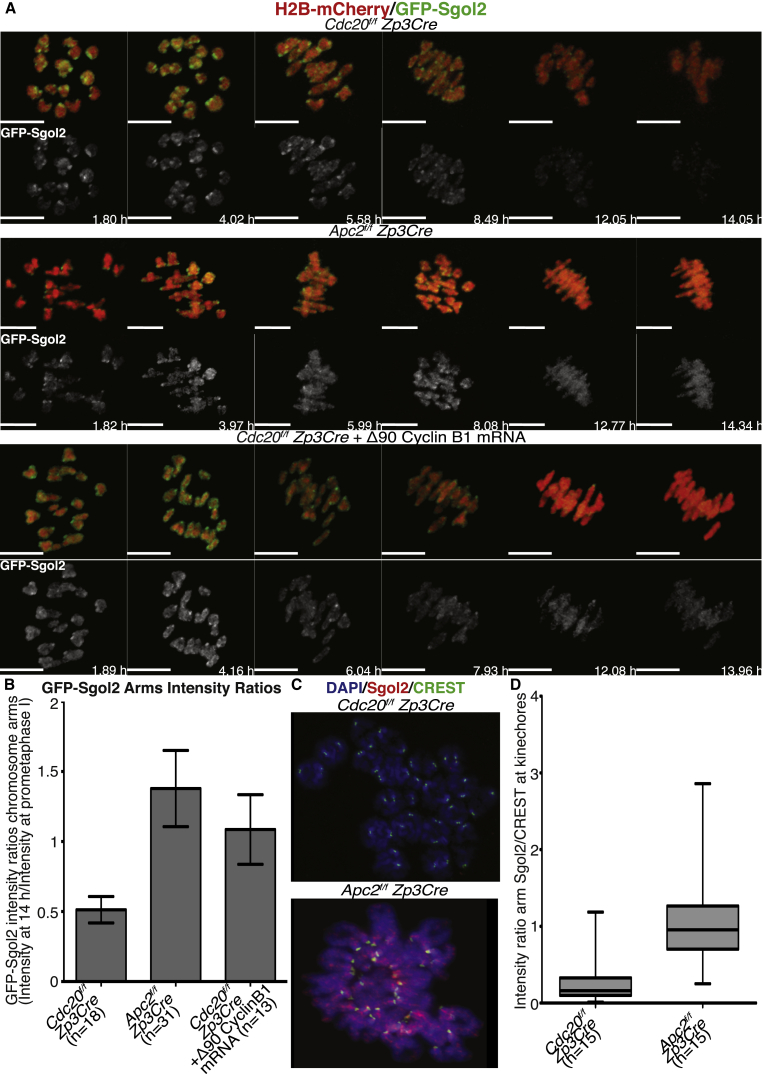
Entry into Meiosis with High Cdk1 Activity Prevents Removal on Sgol2 from Chromosome Arms (A) GV-arrested oocytes harvested from *Apc2*^*f/f*^*Zp3Cre* and *Cdc20*^*f/f*^*Zp3Cre* mice (first and second rows) were microinjected with GFP-Sgol2 (green) and H2B-mCherry (red) or co-injected with GFP-Sgol2 (green), H2B-mCherry (red), and Δ90-cyclin B1 mRNA (third row). After 1 hr in IBMX-containing media, microinjected oocytes were released in IBMX-free M16 medium, and a time-lapse confocal microscopy movie was started. Representative Z-projected time-lapse confocal microscopy images are displayed. Chromosomes and Sgol2 were visualized using H2B-mCherry (red) and GFP-Sgol2 (green), respectively. Times displayed are relative to the time of GVBD. The scale bar represents 10 μm. (B) GFP-Sgol2 intensity signal on chromosome arms at 14 hr was normalized by the GFP-Sgol2 signal intensity on chromosome arms at the prometaphase stage. Normalized intensity values of GFP-Sgol2 signal from *Cdc20*^*f/f*^*Zp3Cre* was compared to normalized GFP-Sgol2 intensities from *Apc2*^*f/f*^*Zp3Cre* (p < 0.0001) and *Cdc20*^*f/f*^*Zp3Cre* microinjected with Δ90-cyclin B1 mRNA (p < 0.0001). Mean and SDs are displayed. The number of oocytes studied is indicated (n). See also Figures S2 and S3. (C) GV-arrested oocytes from *Apc2*^*f/f*^*Zp3Cre* and *Cdc20*^*f/f*^*Zp3Cre* ovaries were released into IBMX-free medium and cultured for 14 hr following GVBD. Chromosome spreads were stained with DAPI (blue), anti-Sgol2 (red), and CREST (green). (D) Fluorescence intensity ratios of Sgol2 on arms and CREST at kinetochores were compared. Compared to *Cdc20*^*f/f*^*Zp3Cre* oocytes, *Apc2*^*f/f*^*Zp3Cre* retained more Sgol2 on chromosome arms at 14 hr post-GVBD (p < 0.0001). Upper and lower bars indicate 95^th^ and 5^th^ percentiles, respectively. The number of oocytes examined is indicated (n).

A curious aspect of chromosome behavior during meiosis I is the localization of the Sgol2 along the arms of bivalent chromosomes after post-GVBD [31, 40]. Sgol2’s relative abundance at arms declines, and it accumulates in the vicinity of kinetochores by the time bivalents co-orient on meiosis I spindles (metaphase I; Figures 2 and S2). To observe these changes in living cells, we imaged chromosomes using time-lapse confocal microscopy following microinjection of mRNAs encoding a GFP-tagged version of Sgol2 into *sgol2*^*Δ/Δ*^ and *Cdc20*^*f/f*^ *Zp3Cre* oocytes, which arrest in metaphase I (Figures 2A, 3A, and S2A). Like endogenous Sgol2, GFP-tagged Sgol2 initially localized along the arms of bivalents. Its subsequent restriction to kinetochores (a process complete between 4 and 8 hr post-GVBD) was accompanied by a decline in its abundance on chromosome arms (Figures 2, 3A, 3B, S2A, and S2B). Association with kinetochores also declined after 8 hr, that is, during the period they arrest in metaphase I due to loss of APC/C^Cdc20^ activity (Figures 3A, 3B, and S2C). Similar results were observed with *Separase*^*f/f*^ *Zp3Cre* oocytes (data not shown).

Surprisingly, GFP-tagged Sgol2 behaved very differently in oocytes lacking Apc2; it persisted on chromosome arms throughout the time course, even 14 hr post-GVBD, by which time it was no longer associated with kinetochores (Figures 3A and 3B). Similar results were observed in Cdh1-depleted oocytes arrested in metaphase I by microinjection of Mad2 mRNA (Figures S3A and S3B). Microinjection of Δ90-cyclin B1 mRNA at GV stage into *Cdc20* knockout oocytes also caused retention of Sgol2 on chromosome arms, suggesting that retention in oocytes lacking Apc2 is due to their elevated Cdk1 activity. Retention of Sgol2 on chromosome arms 14 hr post-GVBD in Apc2-depleted, but not Cdc20-depleted, oocytes was also observed using immunofluorescence staining of endogenous Sgol2 on chromosome spreads (Figures 3C and 3D). Thus, persistent hyper-activation of Cdk1 at GVBD is accompanied by a failure several hours later in dissociation of Sgol2 from chromosome arms.

### Phosphorylation of Sgol2 by Aurora B/C Kinase Is Essential for Sgol2’s Dissociation from Chromosome Arms

**Figure 4:**
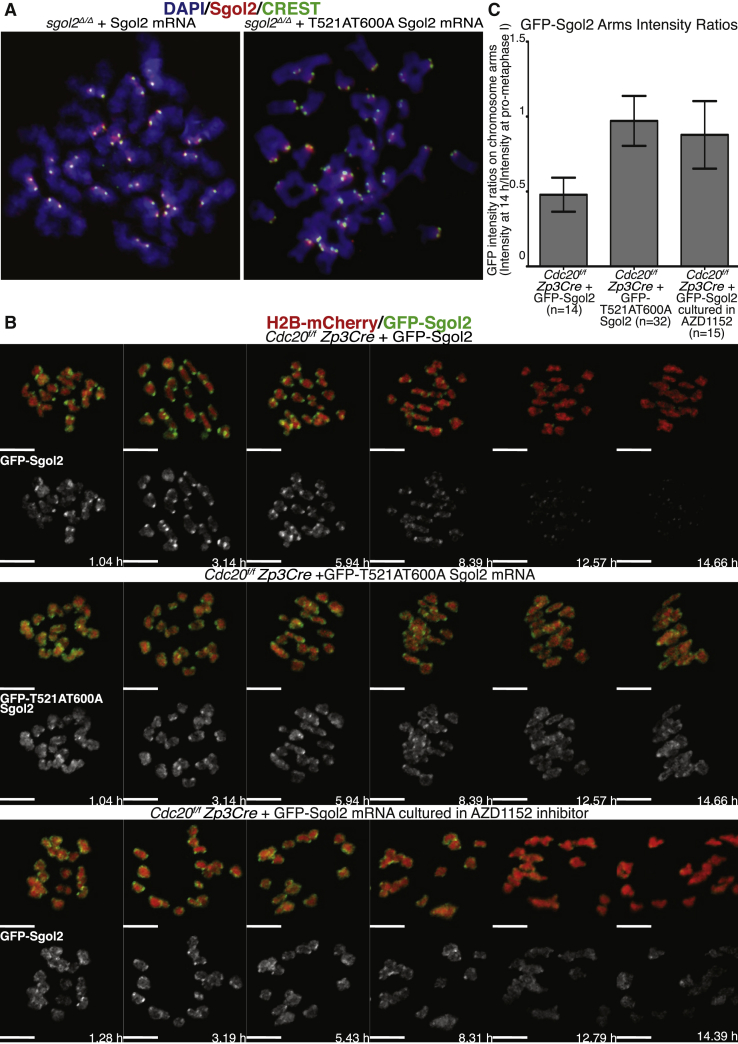
Removal of Sgol2 from Chromosome Arms Requires Aurora-B/C-Mediated Phosphorylation of Sgol2 (A) GV-arrested oocytes harvested from *sgol2*^*Δ/Δ*^ were microinjected with wild-type Sgol2 or T521AT600A Sgol2 mRNA in IBMX-containing media. After 1 hr in IBMX-containing media, oocytes were cultured in M16 medium for 12 hr following GVBD. Chromosome spreads were performed on oocytes that had extruded a polar body and were stained for DAPI (blue), CREST (green), and anti-Sgol2 (red). See also Figure S4. (B) GV-arrested oocytes harvested from *Cdc20*^*f/f*^*Zp3Cre* mice were microinjected with GFP-Sgol2 (green) and H2B-mCherry (red) mRNA (first row) or with GFP-T521AT600A-Sgol2 (green) and H2B-mCherry (red) mRNA (second row). To test whether the Aurora B/C kinase is required for the removal of Sgol2 from chromosome arms, oocytes were cultured in M16 medium supplemented with AZD1152 (100 nM; third row). Representative Z-projected, time-lapse confocal microscope images are displayed. Times displayed are relative to the GVBD. The scale bar represents 10 μm. (C) GFP-Sgol2 intensity on chromosome arms at 14 hr was normalized by GFP-Sgol2 intensity on chromosome arms at the prometaphase stage. *Cdc20* knockout oocytes retained higher levels of GFP-T521A T600A Sgol2 (p < 0.0001) and wild-type GFP-Sgol2 when cultured with Aurora inhibitor (AZD1152; p < 0.0001). Mean and SDs are displayed, and the number of oocytes evaluated is indicated (n).

We have previously reported that microinjection of mRNAs encoding a mutant version of Sgol2 missing Aurora B/C phosphorylation sites (T521A T600A) within two of the four tandem “Sgol2” repeats located between the N-terminal PP2A binding coiled coil and a C-terminal domain involved in centromere recruitment increased PP2A recruitment on the arms (Figure S4A) [31]. Further analysis revealed that the mutant Sgol2 greatly hinders the resolution of chiasmata and thereby causes appreciable levels of non-disjunction at the first meiotic division. In contrast, even high levels of wild-type Sgol2 mRNA have little effect (Figures 4A and S4B) [31]. Live imaging of *Cdc20*^*f/f*^ *Zp3Cre* oocytes microinjected with wild-type or T521A T600A mutant GFP-tagged Sgol2 mRNAs revealed that the latter, but not the former, persisted on chromosome arms, even after 14 hr, which is long after Cdc20 depletion will have prevented the oocytes from undergoing the first meiotic division (Figures 4B and 4C). Consistent with the notion that phosphorylation of T521 and T600 by Aurora B/C kinases is necessary for Sgol2’s dissociation from chromosome arms, incubation of *Cdc20*^*f/f*^ *Zp3Cre* oocytes with AZD1152, a specific Aurora B/C inhibitor, had a similar effect (Figures 4B and 4C).

### APC/C^Cdh1^ Facilitates Aurora B/C Kinase-Dependent Phosphorylation of Sgol2

**Figure 5:**
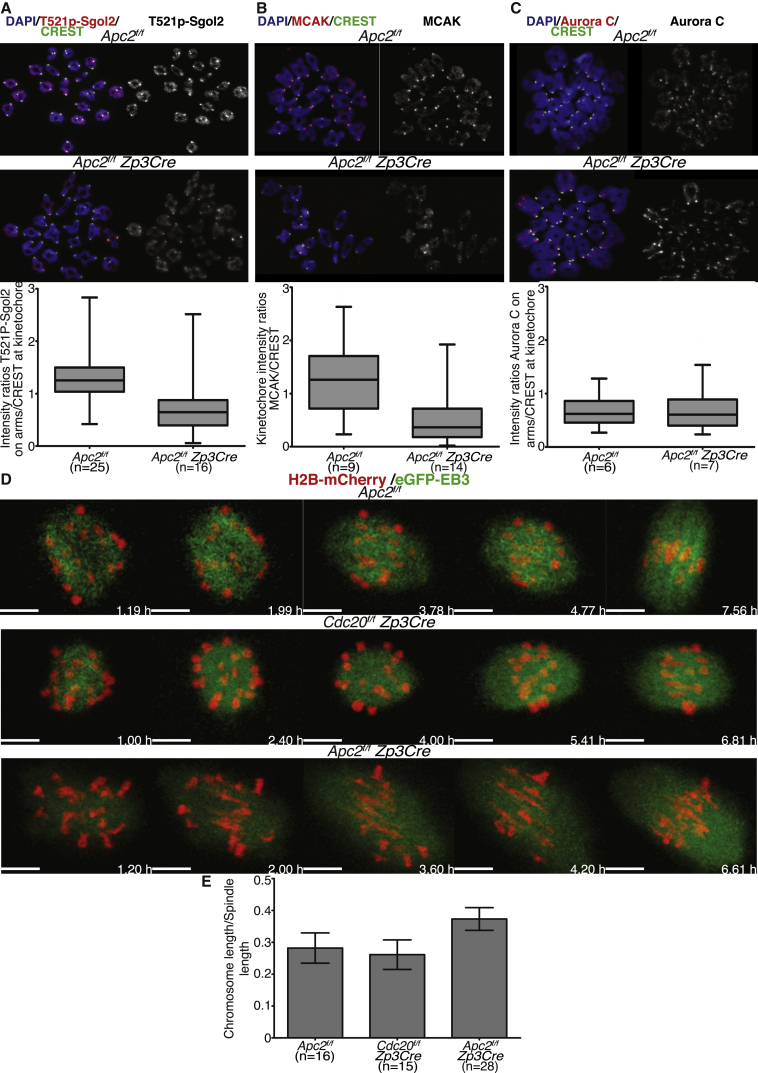
Aurora-B/C-Dependent Phosphorylation of Sgol2 Is Reduced in *Apc2* Knockout Oocytes; Consequently, MCAK Localization Is Reduced and Inter-kinetochore Distance Is Increased in *Apc2* Knockout Oocytes (A) GV-stage oocytes harvested from *Apc2*^*f/f*^ and *Apc2*^*f/f*^*Zp3Cre* ovaries were cultured for 4 hr following GVBD. Chromosome spreads were stained with DAPI (blue), anti-T521p-Sgol2 (red), and CREST (green). Fluorescence intensity ratios of T521p-Sgol2 on chromosome arms and CREST at kinetochores were compared (p < 0.0001). Mean and SDs are displayed, and the number of oocytes examined is indicated (n). (B) Chromosome spreads were prepared at 4 hr post-GVBD. Slides were stained for DNA (blue), MCAK (red), and CREST (green). Fluorescence intensity ratios of MCAK and CREST at kinetochores were compared (p < 0.0001). Mean and SDs are displayed, and the number of oocytes examined is indicated (n). (C) Oocytes were cultured for 4 hr following GVBD in IBMX-free medium. Chromosome spreads were stained with DAPI (blue), CREST (green), and anti-Aurora C (red). Fluorescence intensity ratios of Aurora C on chromosome arms and CREST at kinetochores from *Apc2*^*f/f*^ and *Apc2*^*f/f*^*Zp3Cre* oocytes were compared (p = 0.548). Mean and SDs are displayed, and the number of oocytes evaluated is indicated (n). (D) Oocytes harvested at GV stage from *Apc2*^*f/f*^, *Cdc20*^*f/f*^*Zp3Cre*, and *Apc2*^*f/f*^*Zp3Cre* females were microinjected with H2B-mCherry (red) to visualize chromosomes and EB3-GFP (green) to visualize microtubules. Z-projected (12 Z slices acquired 1.5 μm apart), time-lapsed live-cell confocal images are displayed. (E) Ratios of chromosome length normalized by spindle length were calculated for metaphase I stage oocytes. Normalized chromosome lengths from *Apc2*^*f/f*^ were compared to normalized chromosome lengths from *Cdc20*^*f/f*^*Zp3Cre* (p = 0.2214) and *Apc2*^*f/f*^*Zp3Cre* (p < 0.0001). Mean and SDs are displayed, and the number of oocytes observed is indicated (n). See also Figure S5.

To test whether retention of Sgol2 on chromosome arms in oocytes lacking Apc2 is caused by its reduced phosphorylation, we stained chromosome spreads using an antibody specific for T521 phosphorylation [41]. This revealed that oocytes from *Apc2*^*f/f*^ *Zp3Cre* females had lower levels of Sgol2 T521 phosphorylation along chromosome arms than those from *Apc2*^*f/f*^ littermates at 4 hr post-GVBD (Figure 5A). MCAK’s recruitment to kinetochores, another event known to depend on Sgol2’s phosphorylation by Aurora B/C, was also reduced in oocytes lacking Apc2 (Figure 5B), despite normal levels of this kinase on chromosome arms (Figure 5C). As in the case of oocytes expressing T521A T600A Sgol2 instead of wild-type, the reduction in MCAK at kinetochores was accompanied by stretching of bivalent chromosomes during metaphase in oocytes from *Apc2*^*f/f*^ *Zp3Cre* females, but not those from *Apc2*^*f/f*^ littermates or *Cdc20*^*f/f*^ *Zp3Cre* females (Figure 5D), giving rise to greater distances between maternal and paternal kinetochores in Apc2-deficient oocytes. Likewise, depletion of Apc2, but not that of Cdc20, caused chromosomes to enter the ball phase and to co-orient their kinetochores earlier than wild-type (Figure 5D), as has been found in oocytes expressing high levels of Sgol2 with low Aurora B/C kinase activity [31]. Importantly, similar results were observed in oocytes from *Cdc20*^*f/f*^ *Zp3Cre* females microinjected with Δ90-cyclinB1 mRNA (Figure S5). On the basis of these observations, we suggest that Sgol2’s dissociation from chromosome arms in response to its phosphorylation by Aurora B/C kinase needs APC/C^Cdh1^ to prevent a precipitous rise in Cdk1 activity at the onset of GVBD.

### APC/C^Cdh1^-Dependent Removal of Sgol2 from Chromosome Arms Prevents Chromosome Missegregation at the First Meiotic Division

**Figure 6:**
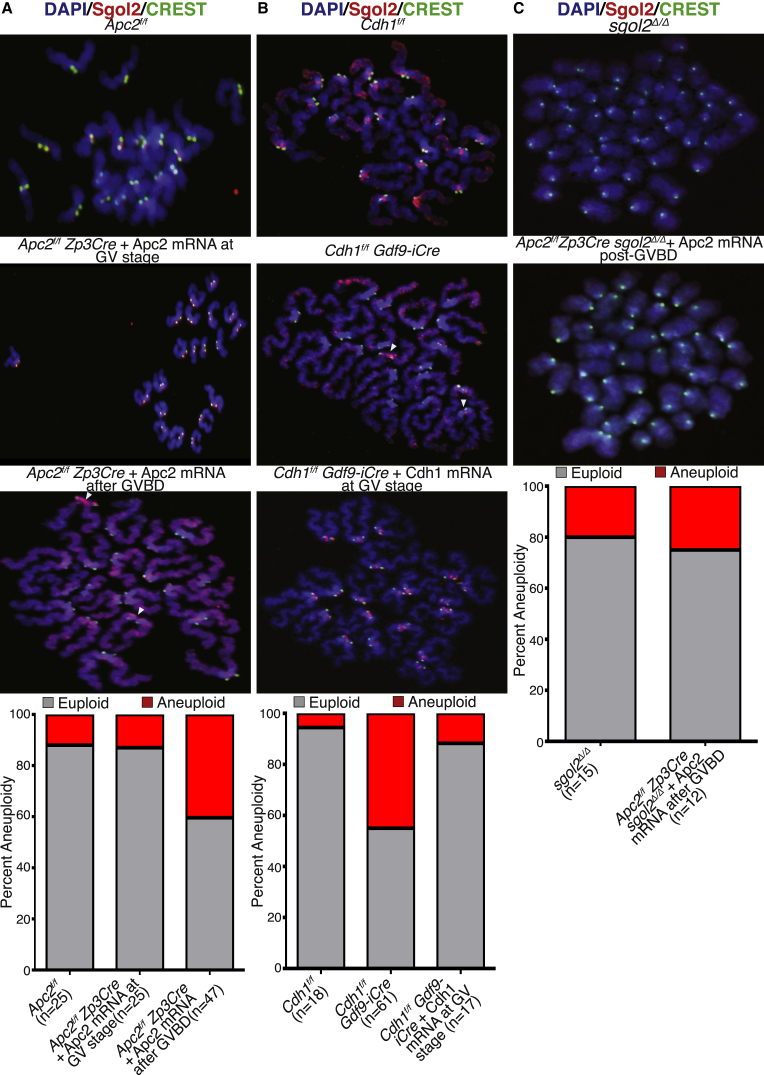
Retention of Sgol2 on Chromosome Arms during Anaphase I Causes Abnormal Attachments and Increases Aneuploidy in *Apc2* and *Cdh1* Knockout Oocytes (A) *Apc2*^*f/f*^ and *Apc2*^*f/f*^*Zp3Cre* oocytes were harvested in IBMX-containing media. To prevent metaphase arrest, *Apc2*^*f/f*^*Zp3Cre* oocytes were microinjected with Apc2 mRNA either at GV stage or after 3 or 4 hr post-GVBD. Oocytes were cultured for 12–14 hr following GVBD, and chromosome spreads were performed on oocytes that had extruded a polar body. Slides were stained with DAPI (blue), CREST (green), and anti-Sgol2 (red). Stacked bar plot indicates the incidence of aneuploidy in each group. The number of oocytes examined is indicated (n). (B) Oocytes harvested from the *Cdh1*^*f/f*^ and *Cdh1*^*f/f*^*Gdf9-iCre* ovaries were cultured in medium supplemented with IBMX. A group of *Cdh1*-deleted oocytes were microinjected with Cdh1 mRNA at the GV stage. Oocytes were matured in the M16 medium for up to 12 hr. Chromosome spreads, prepared from oocytes that had extruded the first polar body, were stained with DAPI (blue), CREST (green), and anti-Sgol2 (red). The frequency of aneuploidy was quantified. The number of oocytes analyzed is indicated by n. (C) Oocytes harvested at the GV stage from *Apc2*^*f/f*^*Zp3Cre sgol2*^*Δ/Δ*^ females were microinjected at 3 or 4 hr post-GVBD with Apc2 mRNA. Chromosome spreads were prepared from metaphase II stage oocytes from *sgol2*^*Δ/Δ*^, and *Apc2*^*f/f*^*Zp3Cre sgol2*^*Δ/Δ*^ microinjected with Apc2 mRNA were stained with DAPI (blue), CREST (green), and anti-Sgol2 (red). The incidence of aneuploidy was quantified. The number of oocytes studied is indicated (n).

Sgol2 protects centromeric cohesion at the first meiotic division by preventing cleavage of Rec8 at this location [31, 32]. Retention of Sgol2 on chromosome arms due to the T521A T600A mutations hinders chiasmata resolution and is accompanied by non-disjunction at meiosis I (Figure 4A). Similarly, loss of Cdh1 is associated with chromosome missegregation at the first meiotic division [37, 42]. Mislocalization of Sgol2 in oocytes lacking either Apc2 or Cdh1 might therefore be expected to have a similar effect. This is difficult to address in the case of *Cdh1*^*f/f*^ *Gdf9-iCre* females because of their ovarian failure and impossible to measure in the case of *Apc2*^*f/f*^ *Zp3Cre* females because a lack of APC/C^Cdc20^ activity causes arrest in metaphase I. However, microinjection of Apc2 mRNA into *Apc2*^*f/f*^ *Zp3Cre* oocytes should restore APC/C^Cdc20^ and thereby anaphase. If mRNAs are injected after GVBD, then the high levels of Cdk1 activity due to the lack of APC/C^Cdh1^ will prevent any activity due to this form of the APC/C and meiosis will proceed with APC/C^Cdc20^ alone. If, however, mRNAs are injected at the GV stage, then they should restore both APC/C^Cdh1^ and APC/C^Cdc20^ activity and meiosis I should take place normally. Aneuploidy measured following the first meiotic division was observed in 12% of *Apc2*^*f/f*^ oocytes (controls) and in 13% of *Apc2*^*f/f*^ *Zp3Cre* oocytes injected with Apc2 mRNA at the GV stage. In contrast, 40% of *Apc2*^*f/f*^ *Zp3Cre* oocytes produced aneuploid eggs when Apc2 mRNA was injected 3 or 4 hr post-GVBD. Features unique to these oocytes were mislocalization of Sgol2, fusion at non-centromeric locations, and precocious splitting of sister centromeres, which often resulted in formation of single chromatids at first meiotic division (Figure 6A). *Cdh1*^*f/f*^ *Gdf9-iCre* oocytes exhibited a similar set of phenotypes (Figure 6B), confirming that they are caused by a lack of APC/C^Cdh1^ activity. Importantly, chromosomes with fusions between non-centromeric regions were rarely if ever produced when Apc2 mRNA was injected into *Apc2*^*f/f*^ *Zp3Cre sgol2*^*Δ/Δ*^ double-knockout oocytes at the prometaphase stage (Figure 6C), implying that this phenotype is caused by the abnormal behavior of Sgol2. As expected, these double-mutant oocytes exhibited the high rates of aneuploidy (25%) characteristic of single *sgol2*^*Δ/Δ*^ mutant oocytes (20%) as well as formation of single chromatids due to the lack of any retention of centromeric cohesion (Figure 6C).

### Mathematical Model of Meiotic Entry

**Figure 7:**
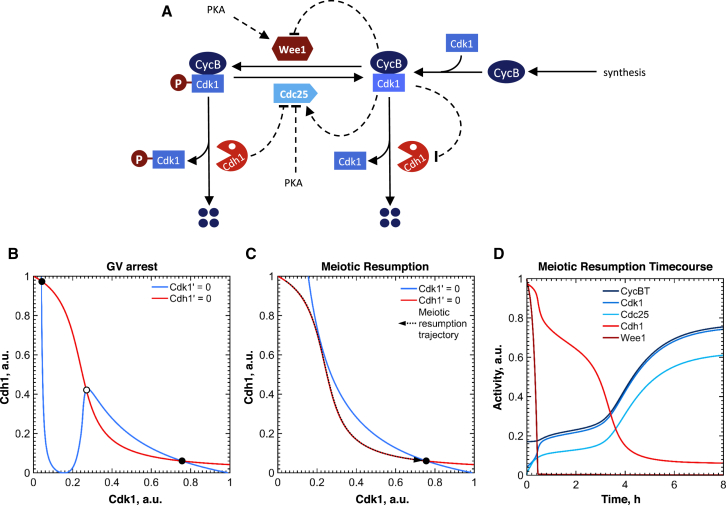
A Model for Meiotic Activation in Mouse Oocytes (A) Model wiring diagram showing interactions between components. Cdk1:CycB activity in early meiosis is determined by APC/C^Cdh1^-mediated CycB degradation and inhibitory phosphorylation of Cdk1 by Wee1, counteracted by Cdc25. Cdk1 inhibits Wee1 and Cdh1 and activates Cdc25, creating positive/double-negative feedback loops. Cdh1 also promotes Cdc25 degradation. (B and C) Phase-plane analysis of the meiotic control network. The steady-state activities (nullclines) of Cdh1 as a function of Cdk1 (red) and Cdk1 as a function of Cdh1 (blue) are plotted for wild-type oocytes at GV arrest (B) and after release from IBMX (C). By definition, intersections of these curves correspond to stable (black circle) or unstable (empty circle) steady states of the whole system. The stable steady state in the upper left corner of (B) corresponds to GV arrest, whereas the one in the bottom right corner corresponds to prometaphase I. At meiotic resumption (C), the upper steady state is lost, leaving only the prometaphase state. The resulting transition to the prometaphase state occurs along a trajectory indicated by the dashed arrow. (D) Time course simulation of the transition described in (C). Species names correspond to the active form of the specified component: i.e., Cdk1 is the number of active (unphosphorylated) CycB:Cdk1 complexes and Cdc25 is the level of active, phosphorylated Cdc25. CycBT is the total of both free and Cdk1-bound CycB pools. Detailed analysis of *Cdh1*, *Cdc25B*, and *Cdh1 Cdc25B* double knockout is presented in Figure S6.

To explore the consequences of the interplay between APC/C^Cdh1^-dependent cyclin degradation and the Cdk1 phosphorylation switch, we created a simple mathematical model (Figure 7A) and analyzed the qualitative dynamics of the system. The steady-state activity of APC/C^Cdh1^ (red curve, Figure 7B) is a sigmoidal decreasing function of cyclin B/Cdk1 because Cdk1 phosphorylation inhibits Cdh1 binding to APC/C [43]. The steady-state activity of Cdk1 (blue curve, Figure 7B) is initially bistable with respect to Cdh1 as a result of double-negative/positive feedback loops between Wee1/Cdc25 and Cdk1. The Cdk1 and Cdh1 steady-state curves intersect to give two stable steady states for the entire system (filled circles): a high-Cdh1, low-Cdk1 GV arrest state (top left) is separated from a low-Cdh1, high-Cdk1 prometaphase state (bottom right) by an intermediate unstable steady state (open circle), which acts as an energy barrier, preventing transition from GV arrest into prometaphase.

Partial inactivation of inhibitory Cdk1 phosphorylation by PDE3A-mediated PKA inhibition [8, 44, 45, 46] upon meiotic resumption shifts the Cdk1 steady-state curve upward, eliminating the GV-arrested state (Figure 7C). The system therefore becomes irreversibly committed toward the other stable “attractor” in the bottom right corner with high Cdk1 and low APC/C^Cdh1^ activities, corresponding to prometaphase.

Time course simulations (Figure 7D) show that, at GVBD, an initial rapid fall in Wee1 and rise in Cdc25 activity allows Cdk1 activity to increase almost to the level of total CycB, as inhibitory tyrosine phosphorylation is lost. However, accumulation of cyclin B and Cdc25B is initially slow, because APC/C^Cdh1^ remains active and continues to delay the transition into prometaphase until it becomes gradually inactivated by cyclin B/Cdk1. Therefore, in contrast to mitosis, inactivation of Cdk1 inhibitory phosphorylation before APC/C^Cdh1^ provides a gradual increase of cyclin B and Cdk1 activity during meiotic resumption. In the absence of APC/C^Cdh1^, Cdk1 activation becomes fast and similar to mitosis, because inhibitory phosphorylation is the only factor holding back Cdk1 activation (Figure S6).

Our model suggests that the regulatory mechanisms controlling cyclin B degradation and Cdk1 activation have different physiological functions during meiotic progression. Partial inactivation of inhibitory Cdk1 phosphorylation results in a small increase in Cdk1 activity, which initiates the slow process of Cdh1 inactivation and cyclin B accumulation. Once Cdh1 has fallen below a threshold level, cyclin B rapidly accumulates, leading to full Cdk1 activation. In this model, partial inactivation of inhibitory phosphorylation acts as a spark, which initiates a slow-burning fuse of cyclin B accumulation, eventually leading to full Cdk1 activity.

## Discussion

Ubiquitinylation, and hence degradation, of proteins by the APC/C depends on their recruitment by a pair of related WD40 repeat proteins called Cdc20 and Cdh1. In addition to having different substrate specificities, Cdc20 and Cdh1 are active at different stages of the cell cycle. Whereas APC/C containing Cdc20 (APC/C^Cdc20^) is active following activation of Cdk1 by the mitotic cyclins A and B, APC/C containing Cdh1 (APC/C^Cdh1^) is usually only active following inactivation of Cdk1 through the destruction of cyclins A and B at the hands of APC/C^Cdc20^ at the onset of anaphase [47]. APC/C^Cdh1^ remains active for most of the subsequent G1 period and prevents accumulation of factors that promote the onset of S phase [48, 49]. Its activity is restricted during G2 and M phases by two mechanisms, namely phosphorylation by Cdk1 and accumulation of an inhibitory chaperone called EMI1 [43, 48, 50, 51]. This inactivity is crucial for the accumulation of mitotic cyclins and hence for entry into M phase. In certain cell types, precocious activation of APC/C^Cdh1^ during G2 phase prevents the accumulation of mitotic cyclins after S phase and thereby has a key role in orchestrating endoreplication instead of mitosis [33, 52, 53].

In mammalian meiosis, APC/C^Cdh1^ regulates levels of cyclins and Cdc25B and thereby delays entry of immature oocytes into meiosis. Because Cdk1 activation requires both binding of cyclins and the Cdc25B-dependent removal of Cdk1 phosphorylation, then how does a wild-type oocyte finally escape APC/C^Cdh1^-dependent G2 arrest? The G2/M transition of mitotic cells, which is equivalent to GVBD in oocytes, has hitherto been thought to be triggered by a rapid rise in Cdk1 activity brought about by switch-like inactivation of inhibitory Cdk1 phosphorylation [36, 54]. However, unlike GV-arrested oocytes, mitotic G2 cells have high cyclin B levels because APC/C^Cdh1^ is switched off much earlier in the cycle (at the G1/S transition) by Cdk phosphorylation [48]. Because during the corresponding GV stage of meiosis both APC/C^Cdh1^ and the Cdk1 phosphorylation are regulating Cdk1 activity, complete activation of Cdk1 activity during meiosis requires inactivation of both of these inhibitions.

The second function of APC/C^Cdh1^ revealed by our studies is equally surprising. APC/C^Cdh1^ ensures that activation of Cdk1 accompanying GVBD is gradual not abrupt. It is also crucial for the subsequent dissociation of Sgol2 from the arms of bivalent chromosomes, a process important for their efficient conversion to dyad chromosomes at the first meiotic division. Because persistence of Sgol2 on the arms of bivalent chromosomes is also observed in oocytes injected with mRNA encoding non-degradable cyclin B1 (Δ90), we suggest that this phenotype is caused by persistent hyper-activation of Cdk1 at GVBD caused by excessive accumulation of cyclin B and not of some other target protein. Nonetheless, we cannot at present exclude the possibility that Δ90-cyclin B1 exerts its effect on Sgol2 removal from chromosome arms by inactivating Cdh1 and thereby blocking degradation of some other key target of APC/C^Cdh1^.

If we are correct that excessive accumulation of cyclin B prior to GVBD is responsible for the subsequent failure of Sgol2 to dissociate from chromosome arms in oocytes lacking APC/C^Cdh1^, then the implication is that the precise kinetics of Cdk1 activation at GVBD are vital for orchestrating the successful segregation of chromosomes at meiosis I. The level of Cdk1 activity rises only gradually during the first few hours following GVBD in wild-type oocytes [5, 6, 7] but jumps immediately to maximal levels in the absence of APC/C^Cdh1^, and we surmise that the window of moderate Cdk1 activity that normally follows GVBD is crucial for Sgol2’s removal from chromosome arms.

Aurora-B-mediated phosphorylation promotes Sgol1, a mitotic paralog of Sgol2, translocation from chromosome arms [55], suggesting that the removal of Sgol2 from chromosome arms might be independent of Sgol2’s interaction with MCAK as Sgol1 does not bind to this motor protein. Moreover, it suggests that Sgol2’s removal might depend directly on its phosphorylation by Aurora B/C kinases. How might excessively rapid activation of Cdk1 interfere with this process? One possibility is that the abrupt rise in the Cdk1 activity at GVBD decreases Aurora B/C kinase activity. Supporting this, we observed the microinjection of non-degradable cyclin B1 reduced phosphorylation of Ser24 on Knl1 (p-Knl1) in wild-type oocytes (data not shown). However, we are not able to rule out the possibility that the abrupt rise in Cdk1 activity might directly affect Sgol2’s interaction with cohesin or other binding partners.

Irrespective of the mechanism, our observations on oocytes lacking APC/C^Cdh1^ reveal that the association of shugoshins with chromosomes is under highly complex spatial and temporal control. In mitotic cells, Sgol1’s association with pericentromeric sequences is thought to depend on prior recruitment to centromeric sequences associated with kinetochores where phosphorylation of histone H2A by Bub1 creates a Sgol1 binding site [56]. A similar phenomenon may pertain also to Sgol2 during meiosis. However, a third pool of Sgol2 exists in oocytes embarking on the first meiotic division, namely one associated with chromosome arms. Because this pool is also associated with PP2A, it must be removed by the time cells activate separase. Two of the deepest mysteries about shugoshins are why they undergo these complex localization events and why they are mediated by such complex regulatory mechanisms.

## STAR★Methods

### Key Resources Table

**Table:** 

REAGENT or RESOURCE	SOURCE	IDENTIFIER
**Antibodies**		

Anti-Centromere Antibodies	Davis Lab, Davis, CA, USA	15-234-0001
Cyclin B1 (D5C10) XP® Rabbit mAb #12231	NEB UK	12231S
Cdc25B Antibody	NEB UK	9525S
Apc2 Antibody	NEB UK	12301S
Purified anti-AURKC mouse monoclonal antibody, clone 10A7	Bethyl Laboratories	A400-022A
Anti-FZR1 antibody	Abcam	ab3242; RRID: AB_2278688
Actin antibody [ACTN05 (C4)]	Abcam	ab3280; RRID: AB_303668
Cdc2 Antibody	NEB UK	9112S
Phospho-cdc2 (Tyr15) Antibody	NEB UK	9111S
anti-Sgol2	This manuscript	NA
Anti-phospho T521 Sgol2	Yoshinori Watanabe [41]	NA
Anti-MCAK	Duane A. Compton [57]	NA
Goat anti-Mouse IgG (H+L) Highly Cross-Adsorbed Secondary Antibody, Alexa Fluor Plus 488	Life Technologies (Invitrogen)	A32723
Goat anti-Mouse IgG (H+L) Highly Cross-Adsorbed Secondary Antibody, Alexa Fluor Plus 647	Life Technologies (Invitrogen)	A32728
Goat anti-Rabbit IgG (H+L) Highly Cross-Adsorbed Secondary Antibody, Alexa Fluor 488	Life Technologies (Invitrogen)	11034
Goat anti-Rabbit IgG (H+L) Highly Cross-Adsorbed Secondary Antibody, Alexa Fluor Plus 647	Life Technologies (Invitrogen)	A32733

**Chemicals, Peptides, and Recombinant Proteins**		

M2 Medium	Sigma	M7167-100ml
IBMX	Sigma	I7018-100mg
1 mL Syringe with needle 26 g x 10mm 1mL (BD 300015), legacy code SZR-190-050B	Fisher Scientific	11754069
EmbryoMax, mod. M16 Medium, Powdered	Miilipores	MR-010P-5F
Mineral Oil	Sigma	M8410-500ml
mMessage mMACHINE T3 Kit	Life Technologies (Ambion)	AM1348
mMessage mMachine T7 Ultra Kit	Life Technologies (Ambion)	AM1345
Poly(A) Tailing Kit	Life Technologies (Ambion)	AM1350
Rneasy Mini Kit (50rxn)	QIAGEN	74104
Nuclease-free water	Life Technologies (Ambion)	AM9938
Poly(vinyl chloride)	Sigma	81388
NuPAGE LDS sample buffer 4x	Life Technologies (Invitrogen)	NP0007
NuPAGE Sample Reducing Agent (10X)	Life Technologies (Invitrogen)	NP0004
NuPAGE Novex 4%-12% Bis-Tris Gel 1.5 mm, 10 well	Life Technologies (Invitrogen)	NP0335BOX
NuPAGE Tris-Acetate SDS Running Buffer (20X)	Life Technologies (Invitrogen)	LA0041
HiMark Pre-Stained High Molecular Weight Protein Standard	Life Technologies (Invitrogen)	LC5699
ECL Prime Western Blotting Detection Reagent	Fisher Scientific (GE Heathcare)	GZ28980926
Immobilon-P Membrane, PVDF	Miilipores	IPVH00010
TWEEN 20	Sigma	P7949-500ML
PRONASE Protease	Merck Chemicals	537088-50KU
Fetal Calf Serum (FCS)	Life Technologies (Invitrogen)	10438018
Normal Goat Serum	Dako UK Ltd	X0907
Paraformaldehyde	Sigma	158127-5G
Kodak Photo-Flo 200 Solution	Amazon	1464510
Histone H1 from calf thymus	Roche Diagnostics Ltd	10223549001
Gamma 32P dATP - 9.25MBq	Perkin Elmer	BLU502Z250UC

**Experimental Models: Organisms/Strains**		

*Cdh1*^*f/f*^	Marcos Malumbres [33]	MGI:3800718
*Cdc20*^*f/f*^	Marcos Malumbres [35]	MGI:4887480
*Cdc25B*^*f/f*^	Helen Piwnica-Worms (The Jackson Laboratory) [58]	MGI:4868694
*Sgol2* targeted mice	Alberto Pendas [32]	NA
*Apc2*^*f/f*^	Kim Nasmyth [34]	MGI:3029825
*Zp3Cre*	Gail Martin (The Jackson Laboratory) [39]	MGI:2176052
*Gdf9-iCre*	Austin J. Cooney [38]	MGI:3056522

**Recombinant DNA**		

pCMV6-GFP-Sgol2	This paper	NA
pCMV6-GFP-T521A T600A Sgol2	This paper	NA
pGEMHE-EB3-mEGFP	Jan Ellenberg [59]	NA
pRNA-H2B-mCherry	Kim Nasmyth [26]	NA

**Software and Algorithms**		

Fiji	[60]	https://fiji.sc
Autofocus Module	[61]	http://www.ellenberg.embl.de/index.php/software/microscopyautomation
T-Coffee	[62]	http://www.tcoffee.org/Projects/tcoffee/
Belvu program	[63]	http://sonnhammer.sbc.su.se/Belvu.html
HMMer2	[64]	http://hmmer.org
Uniprot database	[65]	http://www.uniprot.org
XPPAut		http://www.math.pitt.edu/∼bard/xpp/xpp.html
MATLAB	Mathworks	http://www.mathworks.com
ZEN Blue/Black	Zeiss	https://www.zeiss.com/microscopy/us/products/microscope-software/zen-lite.html
Prism 6.0	GraphPad Software	https://www.graphpad.com/scientific-software/prism/

**Other**		

Corning (430588), 35 mm suspension culture dish	Appleton Woods	BC146
MULTITEST SLIDE, 15 WELL, 4MM, BLUE COATING - PREMIUM	MP Biomedicals UK	096041505E
Retransferpipettes, Blaubrand 2mm, firepolished, ID 108-114 μm	BioMedical Instruments	NA
Cover glass Nunc 4 well chambered glass	Fisher Scientific	TKT-210-030E

### Contact for Reagent and Resource Sharing

Further information and requests for resources and reagents should be directed to and will be fulfilled by the Lead Contact, Kim Nasmyth (ashley.nasmyth@bioch.ox.ac.uk). The pGEMHE-EB3-mEGFP plasmid [59] and *Cdh1*^*f/f*^ [33], *Cdc20*^*f/f*^ [35], *Cdc25B*^*f/f*^ [58], *and Sgol2* targeted lines [32] are covered by MTAs and cannot be transferred by Kim Nasmyth.

### Experimental Model and Subject Details

#### Animal Work

Animals were housed at the Biomedical Sciences Building, University of Oxford, and all procedures were approved by a local Ethical Review Committee and licensed by the Home Office under the Animal (Scientific Procedures) Act 1986.

#### Mouse Strains

Generation of the *Cdh1*^*f/f*^*, Cdc20*^*f/f*^*, Cdc25B*^*f/f*^, and *Apc2*^*f/f*^ targeted mice have been described [33, 34, 35, 58] To create germline specific conditional *Cdh1* knockout females, *Cdh1*^*f/f*^ females were mated with either *Gdf9-iCre* or *Zp3Cre* targeted males [38, 39], and male F1 offspring of the genotype *Cdh1* ^*f/+*^ *Zp3Cre* and *Cdh1*^*f/+*^ *Gdf9-iCre* were crossed with *Cdh1*^*f/f*^ females to obtain germ cell specific conditional knockout females. Similarly, we generated *Cdc25*^*f/f*^ *Gdf9-iCre*, *Apc2*^*f/f*^ *Zp3Cre* and *Cdc20*^*f/f*^ *Zp3Cre* females.

To get *Cdh1*^*f/f*^ *Cdc25B*^*f/f*^ *Gdf9-iCre* females, we crossed *Cdh1*^*f/f*^ *Gdf9-iCre* males with *Cdc25B*^*f/f*^ females to get *Cdh1*^*f/+*^*Cdc25B*^*f/+*^ *Gdf9-iCre* males and *Cdh1*^*f/+*^ *Cdc25B*^*f/+*^ females, which were inter-crossed to get homozygous *Cdh1*^*f/f*^ *Cdc25B*^*f/f*^ *Gdf9-iCre* males and *Cdh1*^*f/f*^ *Cdc25B*^*f/f*^ females. F3 males with genotype *Cdh1*^*f/f*^ *Cdc25B*^*f/f*^ *Gdf9-iCre* were mated to *Cdh1*^*f/f*^ *Cdc25B*^*f/f*^ females to generate females for experiments.

*Sgol2* knockout females were generated as previously reported [32]. To create *Apc2*^*f/f*^ *Zp3Cre sgol2*^*Δ/ Δ*^ females, we crossed *Apc2*^*f/f*^ *Zp3Cre* males with *sgol2* ^*Δ/+*^ females to get *Apc2*^*f/+*^ *Zp3Cre sgol2* ^*Δ/+*^ males and *Apc2*^*f/+*^ *sgol2* ^*Δ/+*^ females. These mice were then inter-crossed to get *Apc2*^*f/f*^ *Zp3Cre sgol2* ^*Δ/+*^ males and *Apc2*^*f/f*^ *sgol2* ^*Δ/+*^, which were mated to generate *Apc2*^*f/f*^ *Zp3Cre sgol2*^*Δ/ Δ*^ females.

### Method Details

#### Isolation, Culture, and Microinjection of Oocytes

Ovaries excised from 6-12 weeks old female mice were placed in M2 medium (Sigma Aldrich) supplemented with 200 μM IBMX (Sigma Aldrich). Oocytes released from ovaries with sterile insulin needles were transferred to IBMX-containing M16 medium under oil and cultured at 37°C and 5% CO2. For microinjections, oocytes were placed in IBMX supplemented M2 medium. After an hour of incubation in M16 supplemented with IBMX, oocytes cultured in IBMX-free M16 medium under oil at 37°C and 5% CO2 for live cell imaging or chromosome spread analysis.

#### Preparation of mRNAs for Microinjection

Depending on the promoter on the plasmid DNA, T3 or T7 Ultra mMESSAGE kits (Ambion) were used to generate capped, poly-A tail containing mRNA. mRNA was purified using an RNase Easy Kit (QIAGEN).

#### Microinjection of mRNA

About 5-10 pL of in-vitro transcribed mRNA at 0.1 mg/mL in RNase-free water (Ambion) was microinjected into mature oocytes in M2 medium (under oil) using a Pneumatic PicoPump (World Precision Instruments). Microinjected oocytes were cultured in M16 medium supplemented with IBMX for 1 hr to enable sufficient expression of microinjected mRNA. Oocytes were then released into IBMX-free M16 medium and maintained at 37°C and 5% CO_2_.

#### Live Cell Confocal Imaging

For live-cell time-lapse confocal microscopy experiments, oocytes were cultured in a PeCon environmental microscope incubator at 37°C and 5% CO_2_. Image acquisition was performed using a Zeiss LSM510 META or Zeiss LSM-780 confocal microscope (Zeiss) confocal microscopes equipped with PC-Apochromat 63x/1.2 NA water immersion and PC-Apochromat 20x/0.8 NA objective lenses; GFP was detected using a 488-nm excitation wavelength and mCherry with 561-nm excitation wavelength. Image stacks of 12-16 slices of 1.5 μm were captured every 10-20 min for 14-16 hr. During live-cell imaging, oocytes were tracked using a macro developed in J. Ellenberg’s laboratory at the EMBL [61].

#### Chromosome Spreads

Chromosome spreads were prepared using techniques previously described [66]. The zona pellucida was removed through placing oocytes in M2 medium containing 10 mg/ml Pronase (Sigma Aldrich) for 5-10 min at 37°C. Oocytes were then transferred to an agar dish containing hypotonic solution (50% Fetal Calf Solution (FCS) in deionised water) for 10 min at 37°C. Subsequently oocytes were fixed in drops of paraformaldehyde solution (1% paraformaldehyde, 0.15% Triton X-100, 3mM dithiothreitol, pH-adjusted using NaOH to achieve final pH of 9.2) on a 15-well glass slide (MP Biomedicals) and incubated overnight in a humidified chamber at room temperature. Slides were then dried for 8-10 hr at room temperature. Slides were subsequently washed twice for 5 min each first in 0.4% Photoflo (Kodak) and then in Phosphate-Buffered Saline (PBS). Processing for immunostaining consisted of three 10 min washes in blocking solution (PBS containing 2 mg/l BSA, 1% Tween 20) before incubating with primary antibodies (as reported below) prepared in blocking solution overnight at 4°C before transfer to 37°C for 1 hr. After three additional 10 min washes in blocking solution, slides were incubated in Alexa 488, 568 and 640 conjugated secondary antibodies (1:500, Invitrogen) in blocking solution for 1-2 hr at room temperature. Slides were then washed three times for 10 min in PBS (1% Tween 20) and 10 min in PBS prior to DAPI staining.

In this study, we used CREST (1:250; Davis Lab, Davis, CA, USA), rabbit anti-Sgol2 (1:50) (raised against epitope previously reported [67]), anti-phospho T521 Sgol2 (1:500, gift from Yoshi Watanabe [41]), anti-MCAK (1:500, gift from Duane A Compton [57]) and anti-Aurora C (1:50, Bethly Laboratories, Montgomery, TX, USA).

#### Western Blotting

Fully grown oocytes at the GV stage were washed in PBS/PVA, lysed in SDS sample buffer, boiled at 90°C for 10 min, snap-frozen and stored at −80°C until further use. For western blotting, samples were thawed on ice and pooled together. Proteins were resolved on 4%–12% Bis-Tris gels (NuPAGE; Invitrogen) and transferred using a semi-dry method onto PVDF membranes (Immobilon-P; Millipore). Following transfer, membranes were blocked for 1 hr at room temperature in blocking solution containing 5% nonfat milk and 0.05% Tween in PBS. After one wash with PBS with 0.05% Tween 20 (PBT), membranes were incubated with antibodies against Cyclin B1 (1:250, Cell Signaling, 12231), Cdk1 (1:200, Cell Signaling, 9116), Phospho-Cdk1 (Tyr15) (1:200, Cell Signaling, 4539), Cdc25B (1:250, Cell Signaling, 9525), Cdh1 (1:200, Abcam, ab3242), Apc2 (1:250, Cell Signaling, 12301) or actin (1:500, Abcam, ab3280) at 4°C for 12 hr. Membranes were washed thrice for 10 min each in PBT solution and incubated with a 1:5000 dilution of horseradish peroxidase conjugated anti-mouse or anti-rabbit antibodies in blocking solution for 2 hr. Following secondary antibody incubation, blots were again washed thrice for 10 min each in PBT solution and developed with the ECL system (Pierce ECL Western Blotting Substrate) according to the manufacturer’s protocols.

#### H1 Kinase Assay

Five oocytes at each of the time points were placed in 1.5 mL Eppendorf tubes containing 10 μL of kinase assay buffer (50mM TrisHCl pH7.5, 10 mM Mgcl2, 1mM DTT). After a gentle spin, samples were snap frozen in liquid nitrogen and then stored at −80°C until further processing.

After removing from −80°C, samples were thawed and centrifuged at 13,000 rpm for 10 min at 4°C. The supernatants were collected in new 1.5ml tubes at 4°C. 1mM ATP, 2 μg Histone H1, and 10 μCi gamma-ATP in a total volume of 10 μL of kinase assay buffer was then added to each of the tubes containing the 10 μL oocyte lysates. Samples were subsequently incubated at 37°C for 30 min. The reaction was terminated by adding 7 μL of SDS-PAGE protein sample buffer and boiling the mixture at 100°C for 10 min. Samples were resolved on 4%–12% Bis-Tris gels (NuPAGE; Invitrogen). After Coomassie staining to visualize the Histone H1, gels were dried and incorporated radioactivity was measured.

#### Data Analysis and Plotting

Live cell confocal and chromosome-spread images were imported into Fiji (ImageJ) software [60]. After background subtraction, intensities were calculated using the ImageJ intensity calculation module. Data were then exported to an Excel sheet and after normalization plotted with Prism 6.0 (GraphPad) software.

For spindle length calculations, multiple measurements were calculated from each of the z-projected images at the metaphase stage. Mean and standard deviations were then plotted using the Prism 6.0 (GraphPad) software.

### Quantification and Statistical Analysis

Fluorescent intensity ratios and spindle length measurements were compared between cases and controls using the two-tailed unpaired Student’s t test with α level of 0.05. All quantified data, except Figures 2B, 2F and 7A–7C, are plotted as mean ± SD. Figures 2B and 2F are cumulative frequency plots and Figures 7A–7C are stacked bar plots. Sample sizes and p values are indicated in the figures and figure legends. We have used n to designate the number of oocytes analyzed for all figures, except for Figure 1A. In Figure 1A, n represents the number of females examined. No statistical method was used to predetermine sample size. Statistical analysis was performed using the Prism 6.0 software.

#### Computational Protein Sequence Analysis

Alignments were produced with T-Coffee [62], using default parameters, slightly refined manually and viewed with the Belvu program [63]. Profiles of the alignments, as global hidden Markov models (HMMs), were generated using HMMer2 [64]. Homologous protein sequences were identified by iterative similarity searches with HMMer2 against the Uniprot database [65, 68]. Repeats in Sgol2 family were identified by iterative similarity searches using HMMer2, including intermediate steps of semi-automatic changes in repeats boundaries, aiming to maximize the number of consecutive non-overlapping repeats per protein sequence.

#### Mathematical Model

An ordinary differential equation (ODE) model was developed based on the Novak and Tyson (1993) model [36] for *Xenopus* oocytes and embryos, with the following modifications. APC/C^Cdc20^ is replaced by APC/C^Cdh1^, which is inhibited by Cdk1-dependent phosphorylation in the same manner as Wee1B. Cdc25B, rather than being present at a constant level, is synthesized at a constant rate and degraded in an APC/C^Cdh1^-dependent manner. The model is defined by six ordinary differential equations (ODEs), which describe the interactions between APC/C^Cdh1^, cyclin B/Cdk1, Wee1B and Cdc25B (henceforth referred to in the context of the model as Cdh1, Cdk1, Wee1 and Cdc25) and four algebraic conservation equations. For analytical purposes, the model was reduced to a two-dimensional form by assuming that the activities of Wee1B and Cdc25B, as well as the levels of cyclin B and Cdc25B, are in pseudo-steady states. This simplified model allowed us to plot phaseplane diagrams showing balance curves of the two dynamic variables (Cdk1 and Cdh1) and to estimate the steady states of the whole control system.

The default parameter set of the model simulates a wild-type GV-arrested state. To initiate GVBD, the Wee1B activation rate and Cdc25B inactivation rate were reduced to 20% of their initial value (VaWee = Vi25 = 0.075), to simulate loss of PKA activity. To simulate knockout situations, the level of the relevant component was reduced to 5% of its wild-type level. (Cdh1T = 0.05; Cdc25: ksc25 = 0.0003). We chose not to set these values to 0 to represent the persistence of residual activity in the cell even after disruption of the targeted gene sequence.

Simulations and phaseplane analysis were performed using the freely available software XPPAut http://www.math.pitt.edu/∼bard/xpp/xpp.html, and plotted in MATLAB. The ‘XPPAut.ode’ file used to run the simulations is given below:

# .ode file for mouse meiosis entry

CycBT’ = ksCycB - (kd1CycB + kd2CycB^∗^Cdh1)^∗^CycBT

Cdk1’ = ksCycB - (kd1CycB + kd2CycB^∗^Cdh1)^∗^Cdk1 - (Vi2CDK^∗^Wee1 + Vi1CDK^∗^Wee1p)^∗^Cdk1 + \

(Va2CDK^∗^Cdc25p + Va1CDK^∗^Cdc25)^∗^Cdk1p

Cdh1’ = VaCdh1^∗^Cdh1p/(JCdh1 + Cdh1p) - ViCdh1^∗^Cdk1^∗^Cdh1/(JCdh1 + Cdh1)

Cdc25T’ = ksc25 - (kd1c25 + kd2c25^∗^Cdh1)^∗^Cdc25T

Cdc25p’ = Va25^∗^Cdk1^∗^Cdc25/(J25 + Cdc25) - Vi25^∗^Cdc25p/(J25 + Cdc25p) - \

(kd1c25 + kd2c25^∗^Cdh1)^∗^Cdc25p

Wee1’ = VaWee^∗^Wee1p/(JWee + Wee1p) - ViWee^∗^Wee1^∗^Cdk1/(JWee + Wee1)

#algebraic mass conservation equations

Cdc25 = Cdc25T - Cdc25p

Wee1p = Wee1T - Wee1

Cdk1p = CycBT - Cdk1

Cdh1p = Cdh1T - Cdh1

#Parameters (for WT GV arrest - note: values have a time unit of mins)

p Va1CDK = 0.015, Va2CDK = 1.5, Vi1CDK = 0.015, Vi2CDK = 0.3

p VaCdh1 = 1.5, ViCdh1 = 6, JCdh1 = 0.15, Cdh1T = 1

p VaWee = 0.375, ViWee = 1.5, Jwee = 0.01, Wee1T = 1

p Va25 = 1.5, Vi25 = 0.375, J25 = 0.01

p ksc25 = 0.015, kd1c25 = 0.015, kd2c25 = 0.15

p ksCycB = 0.015, kd1CycB = 0.015, kd2CycB = 0.075

#Initial Conditions (WT GV arrest)

init CycBT = 0.1701, Cdk1 = 0.04000, Cdh1 = 0.9759, Cdc25p = 0.001675, Wee1 = 0.9981, Cdc25T = 0.09295

# XPP settings

@ XP = t, YP = Cdk1, TOTAL = 480, METH = stiff, XHI = 480, YLO = 0, YHI = 1, BOUND = 1000, dt = 0.1

@ NPLOT = 5, yp1 = CycBT, yp2 = Cdk1, yp3 = Cdh1, yp4 = Cdc25p, yp5 = Wee1

done

## Author Contributions

A.R. and K.N. designed experiments. A.R., R.B.M., K.R., M.B.R., J.G., M.E., E.O., S.O., M.W., and J.M. carried out the experiments. A.R., R.B.M., K.R., M.B.R., J.G., M.E., E.O., and M. Herbert analyzed and interpreted data. A.M.P. and M.M. provided critical mouse strains. M. Hopkins and B.N. constructed the mathematical model. L.S.-P. performed the sequence alignment. K.N. supervised the project. A.R. and K.N. wrote the manuscript. All authors reviewed the manuscript.

## Supplemental Information

Document S1. Figures S1–S6

Document S2. Article plus Supplemental Information
