# Supplementary material for: APC/CCdh1 Enables Removal of Shugoshin-2 from the Arms of Bivalent Chromosomes by Moderating Cyclin-Dependent Kinase Activity
Source: Curr Biol. 2017 May 22;27(10):1462–1476.e5. doi: 10.1016/j.cub.2017.04.023 (PMC5457479; doi:10.1016/j.cub.2017.04.023)
Supplement: Document S1. Figures S1–S6 [file mmc1.pdf]

Current Biology, Volume *27*

Supplemental Information

APC/C^{Cdh1} Enables Removal of Shugoshin-2 from the Arms of Bivalent Chromosomes by Moderating Cyclin-Dependent Kinase Activity

Ahmed Rattani, Randy Ballesteros Mejia, Katherine Roberts, Maurici B. Roig, Jonathan Godwin, Michael Hopkins, Manuel Eguren, Luis Sanchez-Pulido, Elwy Okaz, Sugako Ogushi, Magda Wolna, Jean Metson, Alberto M. Pendás, Marcos Malumbres, Béla Novák, Mary Herbert, and Kim Nasmyth

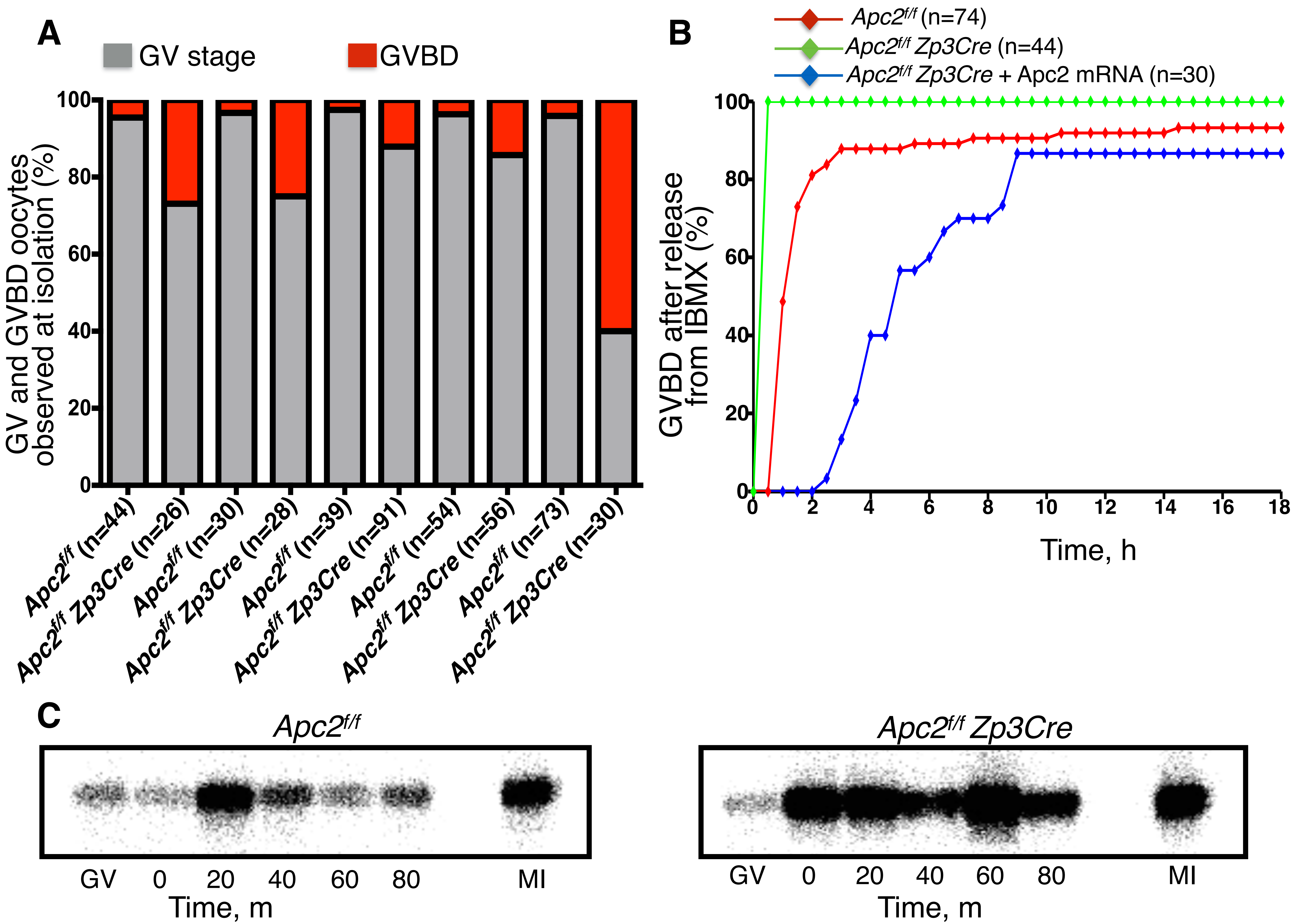

Figure S1. Similar to *Cdh1* knockout, *Apc2* deletion caused premature entry into meiosis and rapid increase in Cdk1 activity at the resumption of meiosis, Related to Figure 1. (A) The plot shows the fraction of GV and GVDB oocytes harvested from *$Apc2^{f/f}$ and $Apc2^{f/f}$ Zp3Cre* females. Five different experiments are displayed. (B) GV stage oocytes, harvested from *$Apc2^{f/f}$ and $Apc2^{f/f}$ Zp3Cre* females in the presence of IBMX, were released into the M16 medium. The plot shows that kinetics of GVBD captured by time-lapse microscopy. (C) Cdk1 activity was estimated using H1 kinase activity. Time course samples -five oocytes for each time point- from each of the indicated group were incubated with radiolabelled ATP and Histone H1. After resolving the sample on SDS-PAGE gel, incorporated radioactivity was imaged. Numbers below each lane indicate minutes after GVBD and MI indicates metaphase I stage.

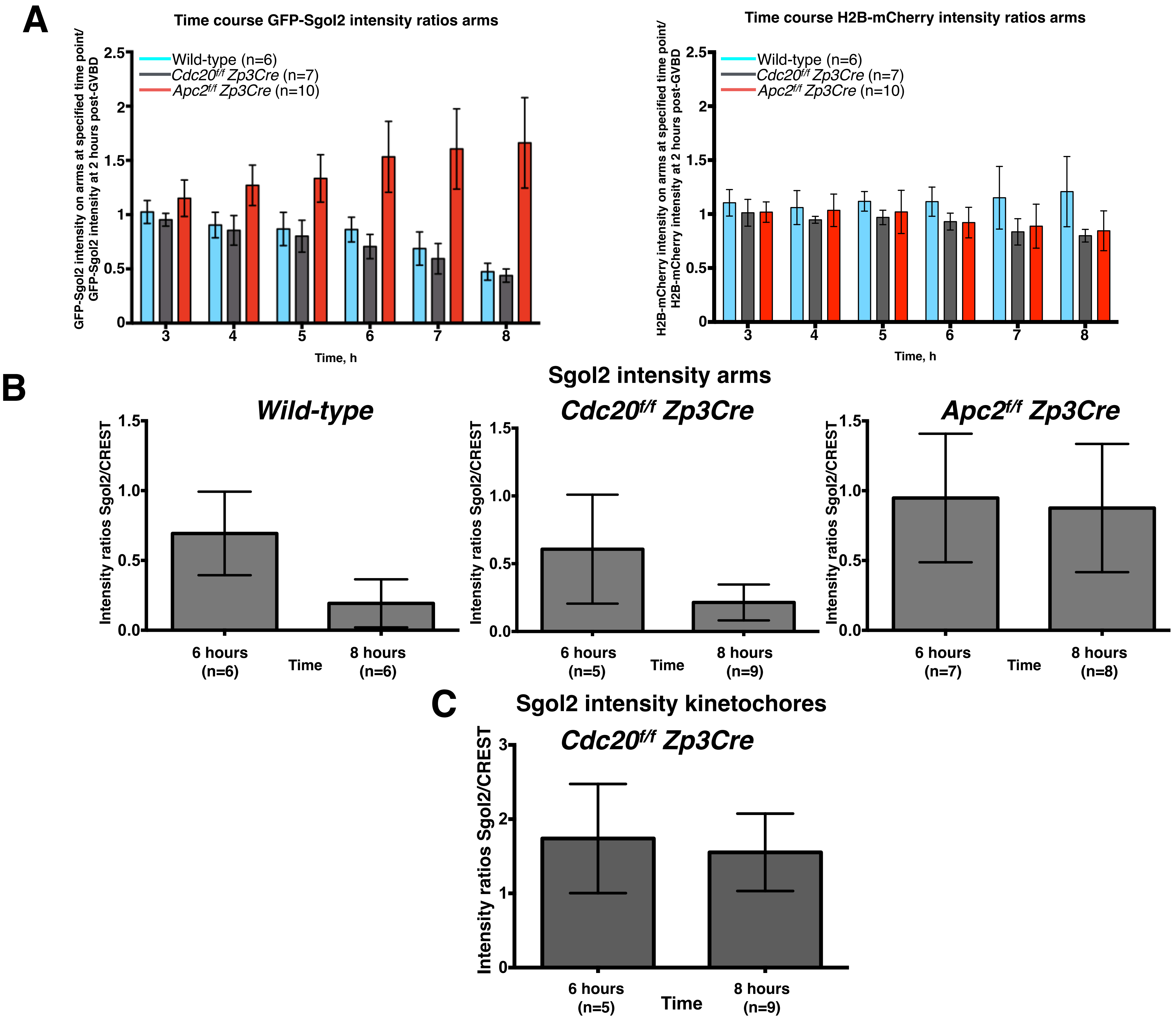

Figure S2: Sgol2 concentration gradually declines from chromosome arms during the prolonged prometaphase and metaphase stages, Related to Figure 2 and 3. (A) Mean GFP-Sgol2 intensities on chromosome arms was calculated and values at each of the indicated time points were normalized to mean GFP-Sgol2 intensity on chromosome arm at 2 hours post-GVBD. The number of oocytes analyzed is indicated (n). (B) The Sgol2 intensities on chromosome arms at indicated time points were normalized to CREST signal at kinetochores. The number of oocytes analyzed is indicated (n). (C) Sgol2 intensities at kinetochores at indicated time points were normalized to CREST signal. The number of oocytes sampled is indicated (n).

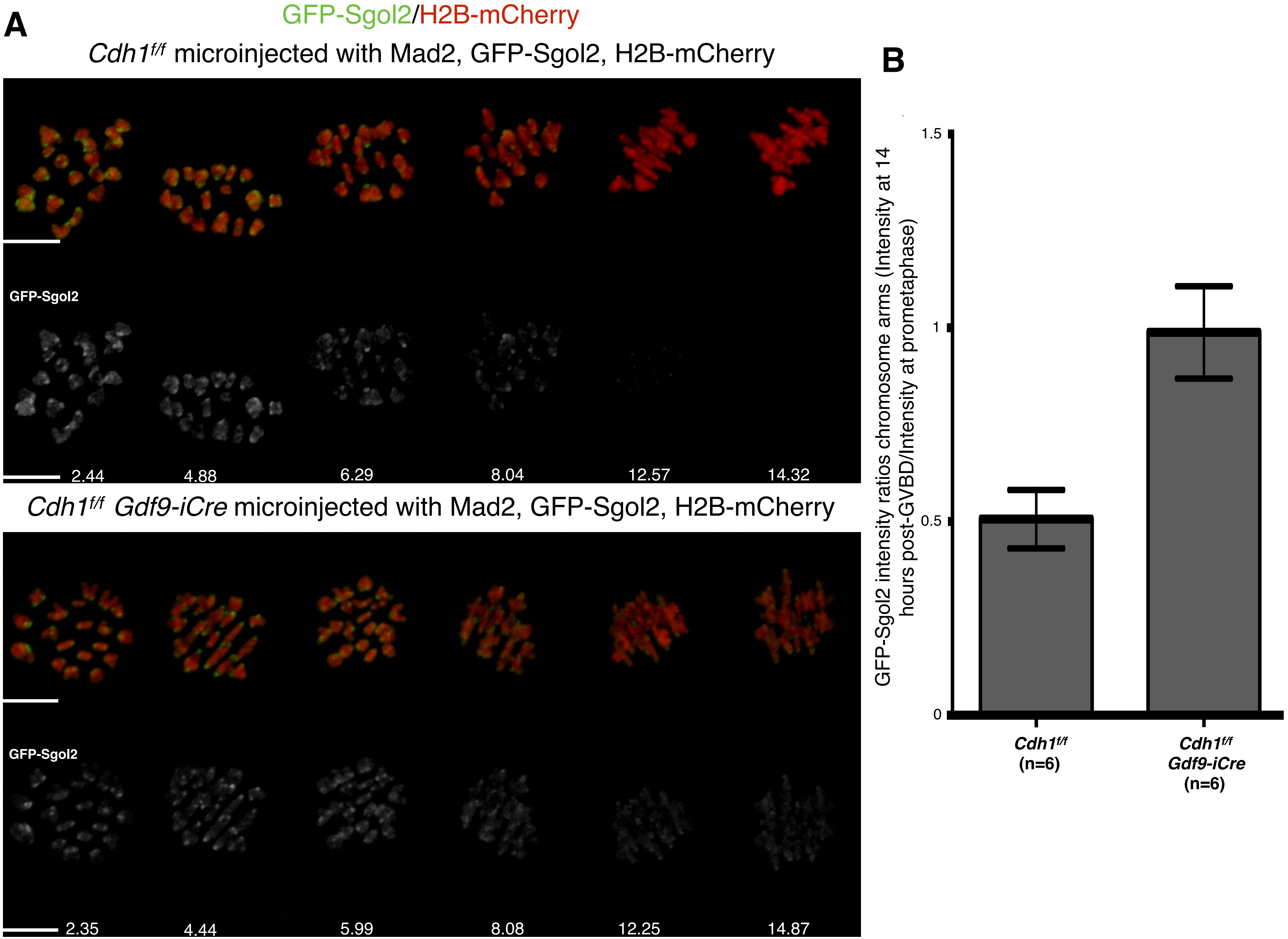

Figure S3: Sgol2 persists on chromosome arms in metaphase arrested *Cdh1* knockout oocytes, Related to Figure 3. (A) *Cdh1f/f* and *Cdh1f/f Gdf9-iCre* oocytes were microinjected with Mad2, GFP-Sgol2 and H2B-mCherry. Live cell time course confocal microscopy images are displayed. (B) Mean GFP-Sgol2 intensities on chromosome arms at 14 hours post-GVBD was normalized to mean GFP-Sgol2 intensity on chromosome arm at 2 hours post-GVBD. The number of oocytes analyzed is indicated (n).

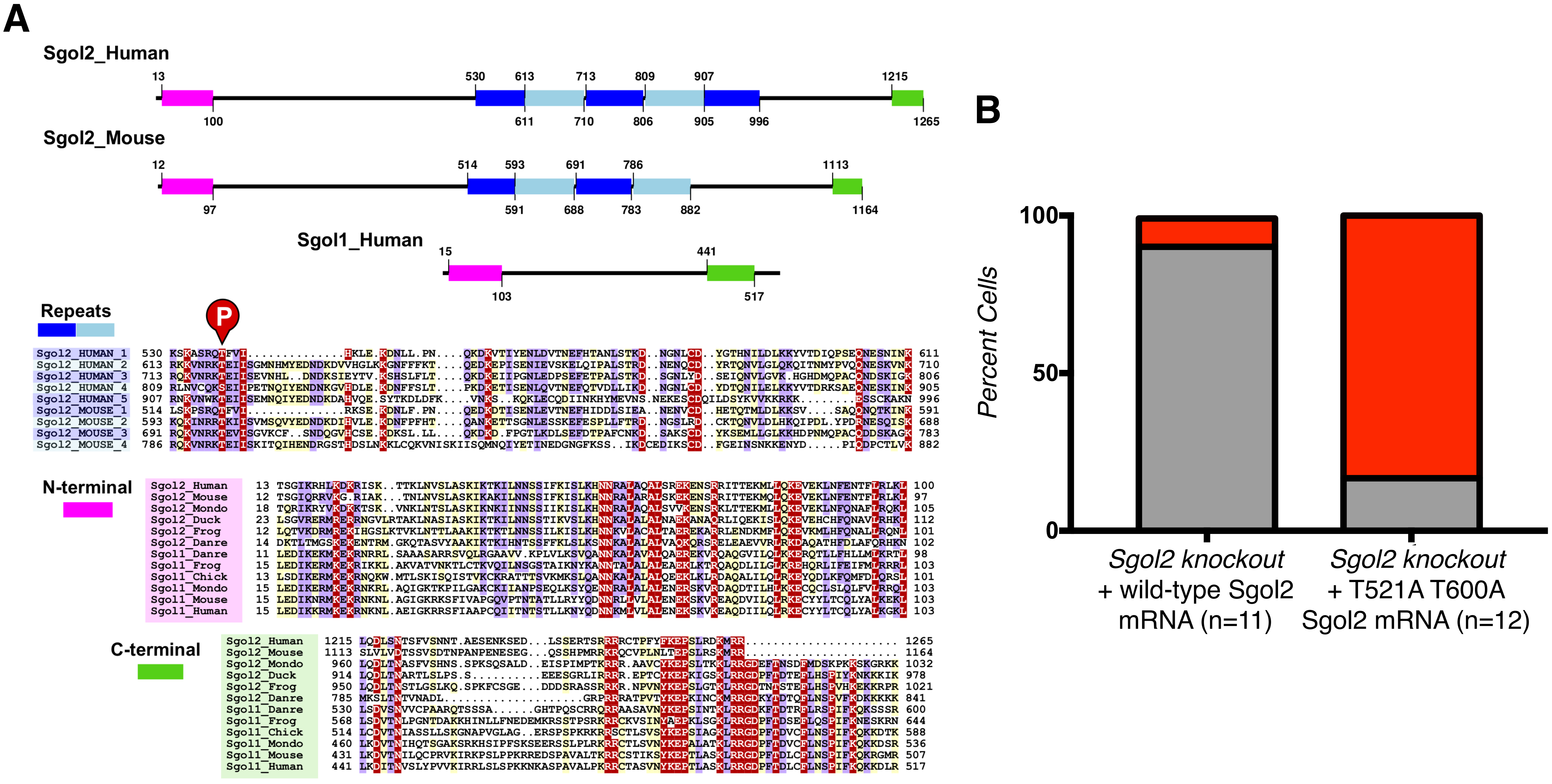

Figure S4: Representations of evolutionarily conserved regions among representative members of Shugoshin family. Sgol2 mutant lacking the Aurora B/C kinase phosphorylation sites causes increased levels of non-disjunction at the first meiotic division, Related to Figure 4 (A) First panel is a multiple sequence alignment of conserved repeats from Sgol2 in human and mouse. Red circle labelled P indicate homologous phosphorylation sites. Second and third panels are representative multiple sequence alignments of amino and carboxyl terminus conserved regions in Shugoshin family, respectively. The amino acid colouring scheme indicates average BLOSUM62 scores (which are correlated with amino acid conservation) for each alignment column: red (greater than 3), violet (between 3 and 1.5) and light yellow (between 1.5 and 0.5). Lanes are named according to (name, database accession, species): Sgol2_Human, UniProt:Q562F6, *Homo sapiens*; Sgol2_Mouse, UniProt:Q7TSY8, *Mus musculus*; Sgol2_Mondo, UniProt:F6PKI2, *Monodelphis domestica*; Sgol2_Duck, GenBank:XP_012952325, *Gallus gallus*; Sgol2_Frog, UniProt:H6X1M8, *Xenopus laevis*; Sgol2_Danre, UniProt:B0UYN0, *Danio rerio*; Sgol1_Human, UniProt:Q5FBB7, *Homo sapiens*; Sgol1_Mouse, UniProt:Q9CXH7, *Mus musculus*; Sgol1_Mondo, UniProt:F7A4C6, *Monodelphis domestica*; Sgol1_Chick, UniProt:E1C2W6, *Gallus gallus*; Sgol1_Frog, UniProt:Q4KLP8, *Xenopus laevis*; Sgol1_Danre, UniProt:A1L1S4, *Danio rerio*. (B) GV stage oocytes harvested from Sgol2 deleted females were microinjected in M2 medium supplemented with IBMX with wild type or T521A T600A Sgol2 mutant mRNA. After one hour of incubation, oocytes were released into IBMX free M16 medium for 12 hours. Chromosome spreads were performed on oocytes that had extruded the first polar body and non-disjunctions was quantified. The number of oocytes analyzed is indicated.

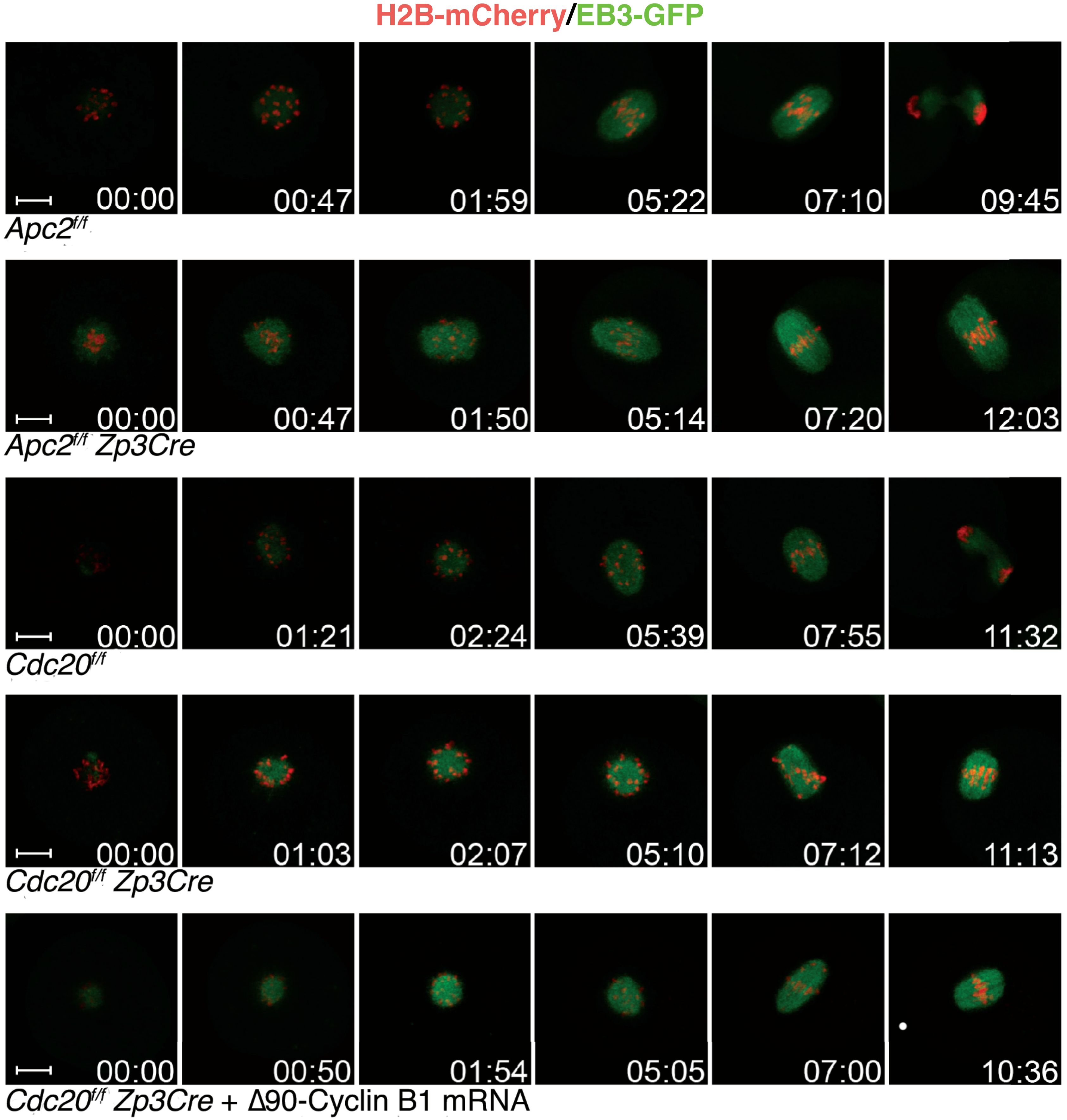

Figure S5: Chromosome alignment and spindle dynamics are altered in *Apc2$^{f/f}$ Zp3Cre* and *Cdc20$^{f/f}$ Zp3Cre* oocytes injected with non-degradable Cyclin B1, Related to Figure 5. GV stage oocytes were microinjected with eGFP-EB3 and H2B-mCherry mRNA (top 4 rows) or co-injected with eGFP-EB3, H2B-mCherry and Δ90-Cyclin B1 mRNA (bottom row). Oocytes were cultured for 1 hour in IBMX-containing media before time-lapse confocal microscopy movie was started. Representative Z-projected time-lapse confocal microscopy images are displayed. Chromosomes were visualised using H2B-mCherry (red), microtubules were visualised using eGFP-EB3 (green). Times displayed are relative to the time of GVBD (00:00). Scale bars represent 16 µm.

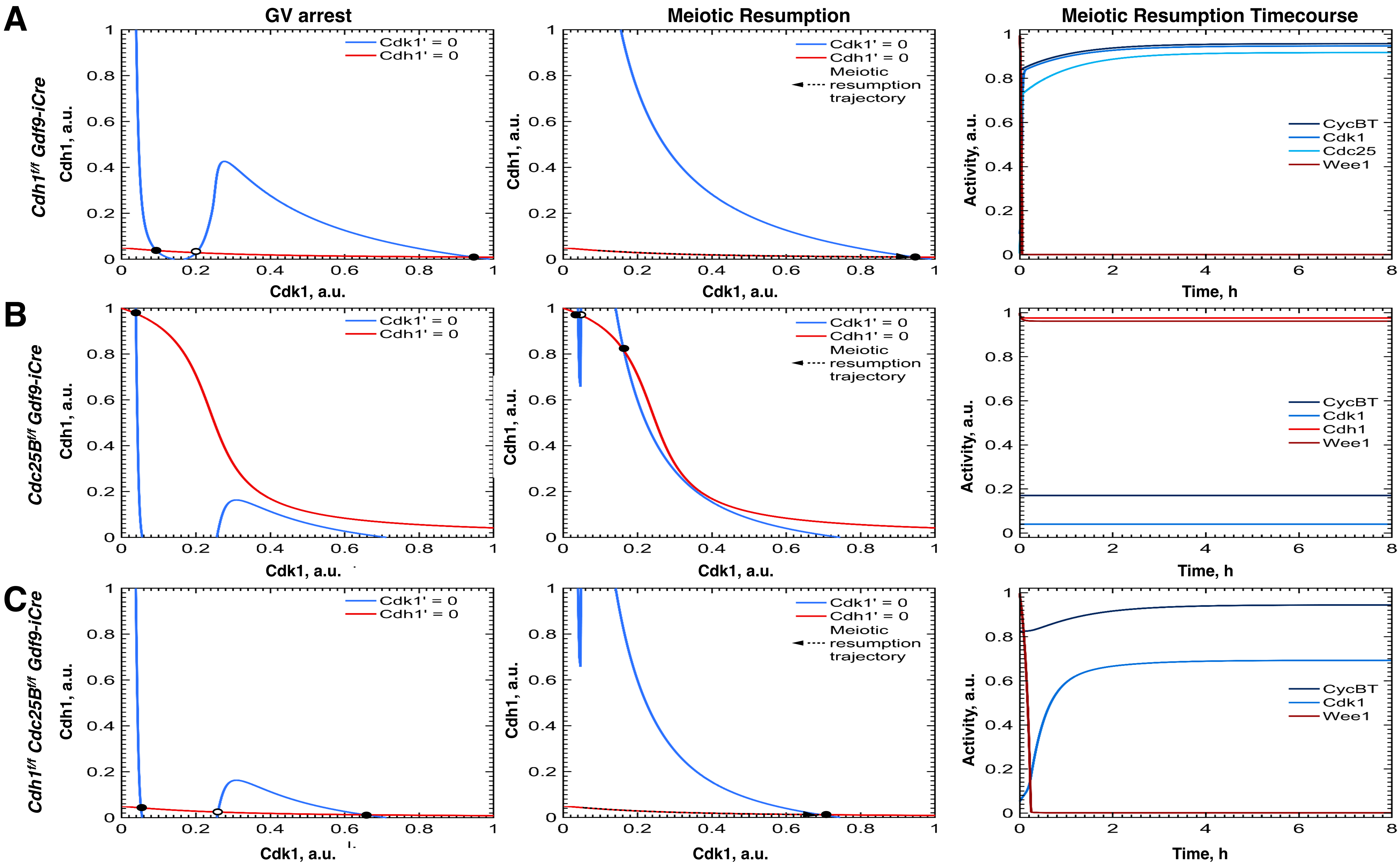

Figure S6: Phase-plane diagrams (first and second column) and time course simulations (third column) for *Cdh1* (A) and *Cdc25B* (B) single knockout and *Cdh1 Cdc25B* double knockout (C) oocytes, Related to Figure 7. The steady state activities of Cdh1 as a function of Cdk1 (red) and Cdk1 as a function of Cdh1 (blue) are plotted for wild-type oocytes at GV arrest (first column) and after release from IBMX (second column). Parameter values used for wild type oocytes at GV arrest are as listed in the XPPAUT code. The following parameter changes correspond to the different situations: (A) *Cdh1* knockout (Cdh1T=0.05), (B) *Cdc25B* knockout (ksc25=0.0003) and (C) *Cdh1 Cdc25B* double knockout (Cdh1T=0.05, ksc25=0.0003). In the second and the third columns both *VaWee* and *Vi25* parameters are set to 0.075 in order to simulate release from IBMX.
